# Approach to Semantic Visual SLAM for Bionic Robots Based on Loop Closure Detection with Combinatorial Graph Entropy in Complex Dynamic Scenes

**DOI:** 10.3390/biomimetics10070446

**Published:** 2025-07-06

**Authors:** Dazheng Wang, Jingwen Luo

**Affiliations:** 1 School of Information Science and Technology, Yunnan Normal University, Kunming 650500, China; 2224100047@ynnu.edu.cn; 2 Engineering Research Center of Computer Vision and Intelligent Control Technology, Department of Education of Yunnan Province, Kunming 650500, China

**Keywords:** complex dynamic scenes, bionic robots, visual SLAM, combinatorial graph entropy, loop closure detection

## Abstract

In complex dynamic environments, the performance of SLAM systems on bionic robots is susceptible to interference from dynamic objects or structural changes in the environment. To address this problem, we propose a semantic visual SLAM (vSLAM) algorithm based on loop closure detection with combinatorial graph entropy. First, in terms of the dynamic feature detection results of YOLOv8-seg, the feature points at the edges of the dynamic object are finely judged by calculating the mean absolute deviation (MAD) of the depth of the pixel points. Then, a high-quality keyframe selection strategy is constructed by combining the semantic information, the average coordinates of the semantic objects, and the degree of variation in the dense region of feature points. Subsequently, the unweighted and weighted graphs of keyframes are constructed according to the distribution of feature points, characterization points, and semantic information, and then a high-performance loop closure detection method based on combinatorial graph entropy is developed. The experimental results show that our loop closure detection approach exhibits higher precision and recall in real scenes compared to the bag-of-words (BoW) model. Compared with ORB-SLAM2, the absolute trajectory accuracy in high-dynamic sequences improved by an average of 97.01%, while the number of extracted keyframes decreased by an average of 61.20%.

## 1. Introduction

The adaptability of natural organisms in complex environments provides inspiration for the development of SLAM technology. Natural organisms can survive and adapt in various environments, and SLAM technology can borrow ideas from this adaptability to continuously improve bionic robots’ adaptability and robustness in different environments, so as to better localize and construct maps in unknown environments. Currently, mainstream SLAM algorithms include radar-based laser SLAM and camera-based visual SLAM (vSLAM). Among them, LiDAR has the characteristics of high accuracy and strong anti-interference ability and has achieved good application results in the fields of 3D imaging [1] and laser SLAM. Due to the low cost of cameras and the ability to obtain richer information in a scene, vSLAM has become a research hotspot [2]. The commonly used cameras in vSLAM include monocular cameras, stereo cameras, and RGB-D cameras, in which the RGB-D camera can simultaneously obtain color and depth information of scene images, i.e., “RGB-D” information. Thus, vSLAM technology based on RGB-D measurement information has been rapidly developed. However, most existing vSLAM schemes are based on the assumption of static scenes [3]. The processing of these methods usually starts with feature extraction on the acquired RGB-D images, followed by inter-frame matching. Then, the successfully matched features are used for pose estimation and further optimized in the backend to obtain the desired localization and mapping results. In this way, some classic SLAM schemes have indeed demonstrated excellent performance in static environments [4,5,6].

In general, the more the map constructed by the SLAM system conforms to the real scene and contains richer information, the more effective it is in assisting robots to operate autonomously in unknown and complex environments and even realizing the cooperative work of multiple robots [7]. But in some practical application scenes, there is often interference from dynamic objects. If the features on these objects are treated as static and used for pose estimation, a large amount of error accumulation will inevitably occur, which will seriously affect the accuracy and stability of the SLAM algorithm [8]. Therefore, vSLAM in dynamic scenes has become a cutting-edge research hotspot in recent years, and many scholars have successively proposed some solutions. Among them, traditional methods [9,10,11,12,13,14] mostly utilize geometric constraints or optical flow-based methods, which can usually meet the real-time requirement but may have a certain impact on the algorithm’s accuracy due to the fewer features in the image or changes in illumination. With the continuous development of deep learning, some researchers have attempted to combine deep-learning-based object detection or instance segmentation models with vSLAM, effectively suppressing the interference of dynamic objects on the SLAM algorithm by removing dynamic objects in the scene through semantic information. Notably, semantic information can help robots better understand the target being operated on, such as achieving precise fruit picking in orchards [15]. Nevertheless, these methods typically require prior information and consume a significant amount of computational resources.

In this work, we introduce a lightweight instance segmentation network, YOLOv8-se,g into the vSLAM algorithm architecture and combine semantic information with the MAD of pixel depth to remove dynamic points to obtain high-confidence static feature points, thereby accurately estimating the camera’s pose. Compared with previous versions, YOLOv8 has achieved a good balance between real-time performance and accuracy [16], which can meet the application requirements of the SLAM algorithm in practical scenes. Herein, YOLOv8-seg provides five models with different speeds and accuracies, which we can choose according to our actual needs. Then, a high-quality keyframe selection strategy is proposed based on semantic information and changes in feature points, and a high-performance loop closure detection strategy based on combinatorial graph entropy is further constructed to improve the accuracy and robustness of loop closure detection. In this way, bionic robots can effectively eliminate pose drift and robustly construct maps with good consistency, thereby better mimicking human behavior and cognitive abilities. Also, these strategies can improve the understanding and adaptability of robots to scenes, thereby enhancing the reliability and safety of autonomous operations in unknown and complex environments, which effectively promotes the development of the robotics industry. The main contributions of this paper are as follows:

1. Based on the segmentation results of YOLOv8-seg, the feature points at the edges of dynamic objects are quickly and accurately judged by calculating the MAD of the depth of the pixel points, which effectively suppresses the influence of dynamic objects on pose estimation.
2. A high-quality keyframe selection strategy is constructed by utilizing the semantic information, the average coordinates of semantic objects, and the degree of change in feature point dense areas.
3. According to the distribution of feature points, representation points and semantic nodes, a graph structure corresponding to the scene structure of the keyframe is constructed, and then the similarity comparison of two keyframes is realized by calculating their graph entropy, effectively improving the accuracy of loop closure detection.
4. A closed-loop similarity calculation method based on scene structure is constructed to determine whether to use only the unweighted graph or both the unweighted graph and the Shannon entropy-based weighted graph, which effectively improves the adaptability of the loop closure detection in different complex scenes.

The rest of the paper is organized as follows. The related works about the bionic robot and the dynamic SLAM algorithms are briefly presented in Section 2. An overview of the proposed framework and methods is introduced in Section 3, and its implementation scheme is detailed. The simulation studies with the public dataset and the experimental testing with a mobile robot are presented in Section 4, while Section 5 concludes the paper and discusses future works.

## 2. Related Work

### 2.1. Bionic Robot

With bionic design, robots can perform more flexible operations in complex environments, exhibiting adaptive abilities and intelligent behaviors similar to natural organisms. For the complex underwater environment, Shi et al. [17] established a kinematic model of the hind legs of Daphnia to provide theoretical support for the design of underwater bionic robots. Tan et al. [18] designed a new type of robotic fish inspired by the long-distance swimming and feeding behavior of suckerfish. Qing et al. [19] proposed a model of beluga whale click column signals, which enables underwater robots to detect different features of targets. Zhu et al. [20] developed a passive roll absorber based on a tuned mass damper to improve the swimming performance of the robot. To improve the adaptability of quadruped robots to uneven ground, Godon et al. [21] designed robot feet imitating ungulates, improving their movement on soft wet ground. Leung et al. [22] constructed a modular motion control scheme based on neural networks using the leg movements of dung beetles. Kalibala et al. [23] proposed a plant-inspired growth robot. Also, creatures with flight capabilities also provide inspiration for the improvement of drones. Shin et al. [24] designed a multi-environment bird-inspired vehicle by simulating a bird jumping for takeoff. Xie et al. [25] designed a UAV landing strategy without distance estimation inspired by flying insects. The proportional multi-degree-of-freedom wing flapping mechanism proposed by Wang et al. [26] makes it possible for aircraft to mimic the flexible and multi-mode wing flapping movements of insects. Furthermore, Liu et al. [27] proposed a multi-strategy improved Siberian Tiger Optimization (MSSTO) algorithm, effectively meeting the task processing requirements of a large amount of remote sensing data. Liang et al. [28] presented an improved macaque optimization algorithm that can generate high-quality paths during drone flight.

It is noteworthy that humans themselves have also provided inspiration for the biomimetic design of robots. Chen et al. [29] proposed an underdriven adaptive function humanoid robotic arm with excellent flexibility, adaptability, and gripping ability. Lapresa et al. [30] developed a method for reproducing touch gestures, which improves the anthropomorphism and safety perception of touch gestures in social robots. The chewing simulator [31] simulates the structure of the human oral cavity, providing a direct validation platform for food experiments. To achieve closed-loop respiratory regulation in vitro for patients with respiratory dysfunction, Zhang et al. [32] designed a bionic soft exoskeleton robot capable of mimicking natural human respiration. In addition to imitating body structure, simulating human perception and understanding of the environment can also improve the performance of robots in complex dynamic environments. Bionic robots, as a cutting-edge field at the intersection of robotics technology and bionics, have shown great potential in the medical, industrial, service, and scientific research fields by simulating the structure, movement patterns, or perception abilities of living organisms. Its development relies not only on technological breakthroughs but also faces multiple challenges such as sports efficiency and environmental adaptability. Therefore, it is necessary to develop more efficient artificial intelligence algorithms and sensor technologies to enhance their environmental perception and decision-making capabilities. Currently, the development of large modeling techniques (e.g., embodied intelligence) provides bionic robots with enhanced environment understanding and decision-making capabilities but is still limited by arithmetic power and model adaptation. Deep learning, utilizing techniques such as Convolutional Neural Networks (CNNs), can endow biomimetic robots with high-precision environmental recognition capabilities, demonstrating outstanding performance in image recognition, object detection, and scene understanding. Taking the field of autonomous driving as an example, deep learning models can accurately recognize road signs, pedestrians, and other vehicles, greatly improving driving safety. This makes the perception of the environment by bionic robots more accurate and reliable in similar scenes. With the support of visual SLAM technology, bionic robots can obtain rich environmental information and use deep learning to simulate the brain’s understanding of different objects in the environment and even determine the position changes of different objects. Hence, by integrating deep learning with SLAM, the bionic robot’s perceptual understanding and adaptive ability in different environments can be effectively improved.

### 2.2. SLAM in Dynamic Scenes

In summary, the vSLAM scheme in dynamic scenes mainly includes traditional methods and deep-learning-based methods. Traditional methods typically utilize geometric methods [9,10,11], filtering [12], and optical flow methods [13,14] to eliminate error accumulation caused by dynamic objects. Islam et al. [10] combined dense optical tracking with spatial scene clustering, and improved the multi-view geometry by matching the pixel point back-projection with the previous frame to achieve the accurate detection of dynamic objects. The principle of the optical flow method is to estimate the pixel motion between two adjacent frames, and some researchers usually improve on the optical flow method for dynamic object recognition [13,14]. Liu et al. [14] recognized dynamic feature points based on a quantitative histogram (QH) and an optical flow angle histogram (FAH) and further proposed a binary segmentation mechanism for the detection of dynamic objects. Traditional SLAM algorithms are simpler to deploy because they do not require pre-training of the model, and traditional methods typically perform well when the scene is highly structured [8]. However, traditional methods rely heavily on the number and quality of features, which can lead to a decrease in the accuracy of the algorithm when affected by factors such as illumination changes and weak textures. Also, due to the lack of semantic information, the calculation basis of traditional methods is relatively simple, and the accuracy of the algorithm will decrease sharply or even fail in highly dynamic scenes with significant scene changes and many dynamic targets.

With the widespread application of deep learning in SLAM, the use of object detection or semantic segmentation networks can effectively solve the interference of dynamic objects. Nevertheless, deep-learning-based methods also have some shortcomings, such as incomplete coverage of dynamic objects by segmentation masks, resulting in incomplete removal of dynamic feature points, and the possibility of missing information in the scene due to vacant areas after removing dynamic objects. Hence, many researchers [33,34,35,36,37,38] have performed further optimization after incorporating deep learning networks into SLAM algorithms. Bescos et al. [33] employed multi-view geometry to detect potential dynamic objects after removing prior dynamic objects and used image projection to patch the blank areas after removing dynamic objects. Wei et al. [34] developed an outlier detection mechanism to mark dynamic feature points after recognizing dynamic objects using a target detection algorithm. Li et al. [35] eliminated potential dynamic points after dynamic elimination using an instance segmentation network by combining the methods of Mahalanobis distance and clustering. Liu and Luo [36] adopted a flood-filling algorithm incorporating edge enhancement within the detection frame to cull dynamic points, thereby obtaining a high-confidence static feature point set. The YOLO-SLAM proposed by Wu et al. [37] exploits a lightweight YOLOv3 with good real-time performance and combines a random sample consensus (RANSAC) algorithm for depth values to avoid the influence of feature points on dynamic objects. Furthermore, many scholars [39,40,41,42] also choose to combine traditional methods with deep learning network models, which complement each other and are also a good solution. However, deep neural networks require a large amount of computing resources, and if complex mathematical methods are chosen to compensate for the network, it will affect the real-time performance of the algorithm. Thus, a concise and efficient method is needed to compensate for the shortcomings of the deep-learning-based approach. Inspired by the above works, we plan to use a lightweight YOLOv8-seg instance segmentation network in the front end to obtain semantic information, which has a faster detection speed compared to the Mask R-CNN network used by Li et al. [35]. Compared with algorithms that only use object detection networks, such as Liu et al.’s algorithm [36], our plan can accurately obtain the specific position of dynamic objects in the image without excessive computation. In addition, we consider adopting the MAD of pixel points for dynamic feature point removal, which is more concise, reduces the consumption of computing resources, and can better ensure the real-time performance of the algorithm.

## 3. Framework and Methods

**Figure 1:**
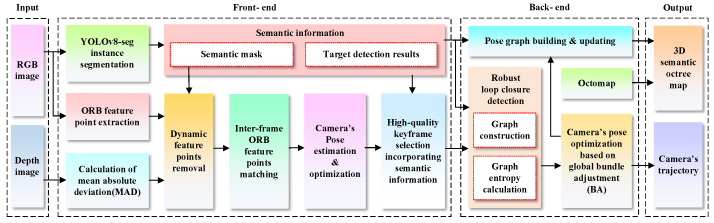
Scheme of semantic vSLAM based on loop closure detection with combinatorial graph entropy in complex dynamic scenes.

The overall architecture of the proposed semantic visual SLAM scheme includes modules such as instance segmentation, dynamic feature removal, keyframe selection, loop detection, and 3D dense map construction, as shown in Figure 1. The system takes RGB images and depth images obtained by a depth camera as inputs, extracts feature points from the RGB images in the front end, and combines YOLOv8-seg for instance segmentation. Meanwhile, according to the segmentation mask and the MAD of depth value, dynamic feature points are accurately removed, and high-quality static feature points are used for inter-frame feature matching. On this basis, to avoid the influence of noise in the depth image as much as possible, we realize the pose estimation by solving the perspective n-point (PnP), which can effectively reduce the influence of the loss of edge depth values on the accuracy of the algorithm. The purpose of the PnP algorithm is to use the minimization of reprojection error for nonlinear optimization. Firstly, the spatial coordinates of a 3D point and its corresponding 2D pixel coordinates are represented using the following expression:

(1) $$d_{i}\left[\begin{array}{c} x_{i}\\ y_{i}\\ 1 \end{array}\right]=KT\left[\begin{array}{c} X_{i}\\ Y_{i}\\ Z_{i}\\ 1 \end{array}\right]$$

where *T* is the Lie algebra representation of the camera rotation matrix *R* and translation *t*, $P_{i}=\left[X_{i},Y_{i},Z_{i}\right]$ denotes the coordinates of the *i*-th 3D spatial point, $d_{i}$ is the depth of $P_{i}$, $p_{i}=\left(x_{i},y_{i}\right)$ denotes the pixel coordinates of the image corresponding to $P_{i}$, and *K* is the internal parameter of the camera.

(2) $$T=\arg\min_{T}\frac{1}{2}\sum_{i=1}^{n}{∥p_{i}^{2D}-\frac{1}{d_{i}}KTP_{i}^{3D}∥}_{2}^{2}$$

In addition, to reduce the redundancy of keyframes and avoid excessive loss of image information caused by removing dynamic points, we comprehensively utilize the semantic information provided by YOLOv8-seg, the average coordinates of semantic objects, and the degree of change in feature point dense areas to select high-quality keyframes. In the back-end, we construct unweighted and weighted maps based on keyframes and utilize combinatorial graph entropy for loop closure detection, which in turn performs global BA optimization of the pose and generates a 3D sparse map. Since the sparse map cannot effectively represent the detailed scene information and has a low visualization degree, we construct a 3D dense map by introducing the Point Cloud Library (PCL). Meanwhile, we project the acquired static feature point cloud into 3D space for each keyframe and assign the point cloud colors to generate local semantic maps according to the semantic information provided by YOLOv8-seg and then splice the local semantic maps constructed by each keyframe according to the pose of the keyframes to generate the final global 3D dense semantic point cloud map. Considering that the density of the point cloud increases as the camera continues to observe and is also affected by noise or outliers, it is inevitable that an invalid point cloud will be generated. Therefore, we further optimize the point cloud using voxel filtering. To allow the generated map to be used for navigation and obstacle avoidance, we also used Octomap to convert the 3D dense point cloud map into an octree map. In the process of filling an octree map based on the map information of a 3D dense point cloud, the influence of observation errors can cause a certain node in the octree map to not be in a fixed “occupied” or “free” state during filling. Thus, a probability logarithm is introduced to represent whether there is an entity at that node, i.e.,

(3) $$L\left(Node|\kappa_{1:t+1}\right)=L\left(Node|\kappa_{1:t+1}\right)+L\left(Node|\kappa_{t}\right)$$

where Node denotes a node in the octree map and $\kappa_{t}$ is the observation at time *t*. When the probability logarithm is greater than a certain threshold, it is considered that the node exists as an entity, and the node is semantically filled to obtain a 3D semantic octree map that can be used for navigation. In what follows, we will describe the main work of this paper in detail.

### 3.1. Dynamic Feature Elimination

**Figure 2:**
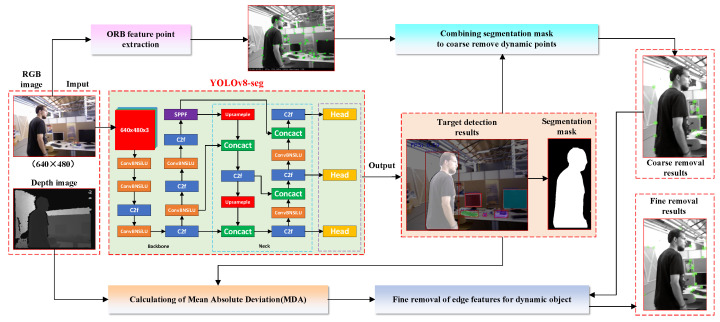
Scenarios for the dynamic feature point elimination using YOLOv8-seg.

At present, YOLOv8-seg is one of the most advanced instance segmentation models; it replaces the C3 module in YOLOv5 with the C2f module to make the model more lightweight, and adopts anchor-free detection heads to adapt to detection objects of different shapes and sizes, thus achieving a good balance between detection accuracy and running speed. Also, the YOLOv8-seg instance segmentation network includes five models, n, s, m, l, and x, in which the n-model is the lightest, while the x-model has higher accuracy, which allows us to make reasonable choices in terms of different applications and thus is more convenient and flexible. In this work, considering that the SLAM algorithm needs to possess good real-time performance to cope with different scenarios, we adopt the n-model of the YOLOv8-seg to detect the dynamic objects in the acquired RGB-D images (640 × 480) and eliminate their dynamic feature points and then utilize the static feature points with high confidence to complete pose estimation. The entire process is shown in Figure 2. To more efficiently use the segmentation mask provided by YOLOv8-seg to detect and eliminate dynamic feature points, we leverage a white mask to cover dynamic objects for identification, namely, if the RGB color value of the mask at the pixel coordinate of the *i*-th feature point is (255,255,255), it is considered as a dynamic feature point and needs to be eliminated. However, it is often difficult for lightweight networks to provide accurate detection results, especially when the dynamic objects are moving too fast or the magnitude of the camera’s movement is large, resulting in blurry extracted scene images. In this case, the mask cannot accurately cover the dynamic objects, especially the edges, leading to incomplete segmentation of the dynamic objects. Additionally, in some application scenarios, segmentation networks may not be able to accurately identify certain regions of dynamic targets (e.g., pedestrians’ feet), resulting in the omission of some dynamic feature points that have not been removed from the static background. Therefore, we combine the depth image with the segmentation mask generated by YOLOv8-seg to calculate the mean absolute deviation (MAD) of the depth of each dynamic object in the image to further accurately detect these “missed segmentation” feature points.

**Figure 3:**
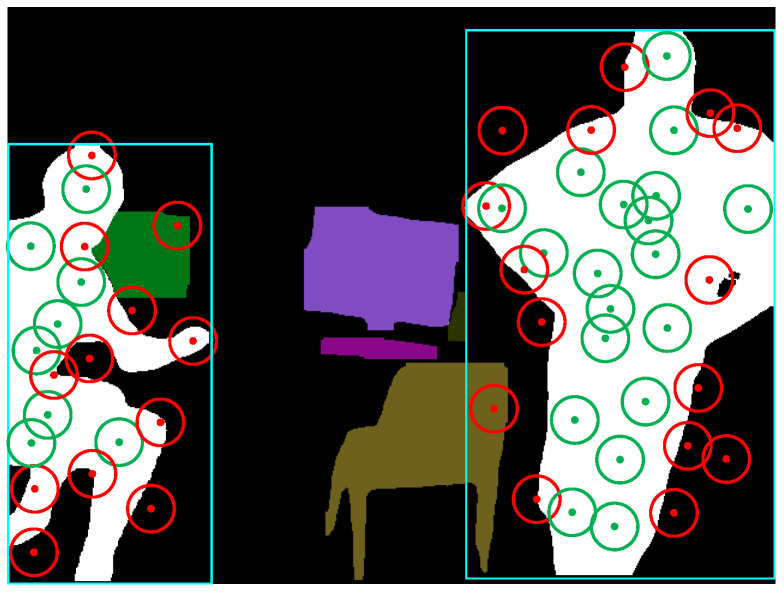
Valid and invalid points sampled in different dynamic mask detection boxes. The green and red dots indicate valid and invalid points, respectively, while the circle indicates a circular range with a radius of 6 pixels.

Specifically, it involves randomly sampling *n* pixels within a dynamic mask and using their depth values to calculate MAD. Since the segmentation mask generated by YOLOv8-seg may possess more or fewer errors compared to real objects, if the depth values of the pixels in the error region are also involved in the calculation of MAD, the actual depth values of the dynamic objects will not be accurately measured. To avoid this situation, we take the center of the detection box of a dynamic object as the basis and generate *n* random numbers inside the box to find *n* pixels, i.e.,

(4) $$p_{\mathrm{pixel}}^{i}=\left(x_{box}+\mathrm{rand}(x_{i}),y_{box}+\mathrm{rand}(y_{i})\right)$$

(5) $$\mathrm{rand}(x_{i})\in \left(-\frac{l_{x}}{2},+\frac{l_{x}}{2}\right),\mathrm{rand}(y_{i})\in \left(-\frac{l_{y}}{2},+\frac{l_{y}}{2}\right)$$

where $l_{x}$,$l_{y}$ and $\left(x_{box},y_{box}\right)$ denote the length, width and center point coordinates of the dynamic object detection box, respectively; $\mathrm{rand}(x_{i})$ and $\mathrm{rand}(y_{i})$ denote the random numbers generated in the x-direction and y-direction, respectively,; and $p_{\mathrm{pixel}}^{i}$ denotes the pixel point corresponding to the *i*-th random number, i∈[1,n]. If the *i*-th pixel point $p_{\mathrm{pixel}}^{i}$ is located on a dynamic mask and its circular range with a radius of 6 pixels all belongs to the region of that dynamic mask, then the depth value of that pixel point needs to be involved in the calculation of the MAD for that dynamic mask and is called a valid point. If there is a portion of its circular range outside of the mask region, the depth value of that pixel point is excluded from the MAD calculation and is called an invalid point, as shown in Figure 3.

Assuming that there are *k* dynamic objects in the image, the mean absolute deviation $MAD_{d}^{j}$ of the depth value of the *j*-th dynamic object is calculated as follows:

(6) $$MAD_{d}^{j}=\frac{1}{n}\sum_{i=1}^{n}\left|d_{i}^{j}-\bar{d^{j}}\right|,j\in \left[1,k\right]$$

(7) $$\bar{d^{j}}=\frac{1}{n}\sum_{i=1}^{n}d_{i}^{j}$$

where $d_{i}^{j}$ denotes the depth value of the *i*-th pixel on the *j*-th dynamic object, and $\bar{d^{j}}$ denotes the average of the depths of the *n* pixel points selected on the *j*-th dynamic object.

Subsequently, in terms of the pixel coordinates of the feature points in the image and the position of the detection box, further judgment is made on the feature points within the detection box. If the detection box of the *j*-th dynamic object contains *m* feature points $p_{f}^{i,j}$,i∈[1,m], then the absolute deviation of the depth value of $p_{f}^{i,j}$ and the average depth value $\bar{d^{j}}$ of the dynamic objects belonging to that detection box is calculated as follows:

(8) $$Dev_{p_{f}^{i,j}}=\left|d_{p_{f}^{i,j}}-\bar{d^{j}}\right|$$

where $d_{p_{f}^{i,j}}$ denotes the depth value of $p_{f}^{i,j}$. If $Dev_{p_{f}^{i,j}}<MAD_{d}^{j}$, it is considered that the feature point $p_{f}^{i,j}$ has the same depth value as the current dynamic object, and $p_{f}^{i,j}$ can be determined as a dynamic feature point and needs to be eliminated.

**Figure 4:**
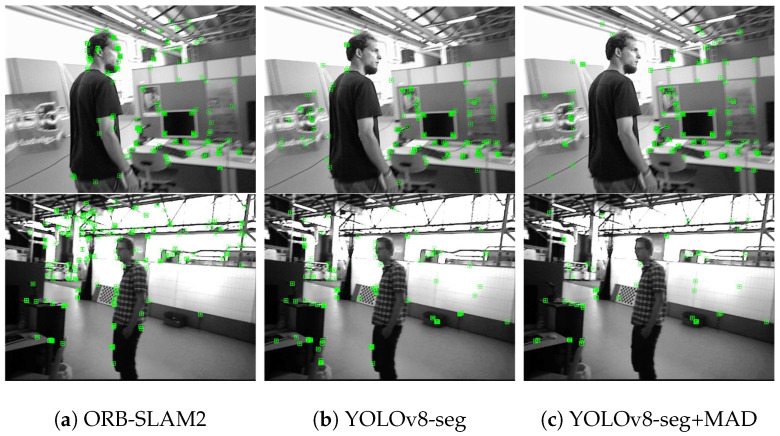
Feature point extraction results of different algorithms. In the top row, about 37 dynamic points in ORB-SLAM2 were involved in the matching; when dynamic culling was performed using YOLOv8-seg, only 8 dynamic points were involved; and when further combined with MAD, only one dynamic point was involved. In the bottom row, about 22 dynamic points in ORB-SLAM2 were involved in the matching; when dynamic culling was performed using YOLOv8-seg, only 9 dynamic points were involved; and when further combined with MAD, no dynamic points were involved.

Figure 4 demonstrates the effects of feature point extraction using different algorithms on the walking_rpy sequence of the *TUM* dataset. Obviously, ORB-SLAM2 extracted a large number of feature points on humans, while using only the segmentation mask provided by YOLOv8-seg can remove most of the dynamic feature points, but the segmentation mask cannot accurately cover dynamic objects, resulting in residual dynamic feature points on the edges of the person that are not eliminated, and these “missed segmentation” feature points will have a negative impact on localization accuracy. In contrast, our method combines the YOLOv8-seg segmentation mask and MAD with pixel depth values to accurately recognize and eliminate feature points at the edges of a person, effectively suppressing the impact of dynamic objects on the accuracy and stability of the algorithm.

### 3.2. High-Quality Keyframe Selection

For the application requirements of complex dynamic scenes, according to the distribution of extracted feature points and semantic entities in the scene image, we propose a high-quality keyframe selection strategy based on patch similarity. First, starting from the first frame, consider every *k* consecutive frames as an interval, and then select candidate keyframes within that interval. Then, each frame is divided into n×m rectangular patches, and the feature point distribution vectors of all patches in the current frame are calculated as follows:

(9) $$Z_{cur}=\left(z_{cur}^{0},z_{cur}^{1},...,z_{cur}^{10+}\right)$$

In Equation (9), if i∈[0,9], then $z_{cur}^{i}$ refers to the number of patches with a feature point count greater than 2i and less than or equal to 2(i+1), while $z_{cur}^{10+}$ denotes the number of patches with a feature point count greater than 20. Likewise, the feature point distribution vector of the reference keyframe is as follows:

(10) $$Z_{ref}=\left(z_{ref}^{0},z_{ref}^{1},...,z_{ref}^{10+}\right)$$

Further, the cosine similarity between $Z_{cur}$ and $Z_{ref}$ is calculated as follows:

(11) $$S_{cos}^{z}\left(Z_{cur},Z_{ref}\right)=\frac{Z_{cur}\cdot Z_{ref}}{∥Z_{cur}∥\cdot ∥Z_{ref}∥}$$

If $S_{cos}^{z}\left(Z_{cur},Z_{ref}\right)$ is less than a certain threshold $S_{th}^{z}$, the frame is considered a candidate keyframe. In this way, *w* candidate keyframes can be obtained, and w<k. On this basis, calculate the number of feature points in each patch and identify the num dense patches with the highest number of feature points. Then, further calculate the average coordinates of num dense patches in the current frame, i.e.,

(12) $$Z_{cur}^{avg}=\left(\alpha_{cur}^{avg},\beta_{cur}^{avg}\right)$$

(13) $$\alpha_{cur}^{avg}=\frac{\sum_{i=1}^{num}\alpha_{cur}^{i}}{num},\beta_{cur}^{avg}=\frac{\sum_{i=1}^{num}\beta_{cur}^{i}}{num}$$

where $\alpha_{cur}^{i}$ and $\beta_{cur}^{i}$ denote the midpoint coordinate values of the *i*-th dense patch, and i∈[1,num]. Moreover, the average coordinates $Z_{ref}^{avg}=\left(\alpha_{ref}^{avg},\beta_{ref}^{avg}\right)$ of each dense patch are calculated in the reference keyframe, and the Euclidean distance between $Z_{cur}^{avg}$ and $Z_{ref}^{avg}$ is calculated as follows:

(14) $$D_{fp}=\sqrt{{\left(\alpha_{cur}^{avg}-\alpha_{ref}^{avg}\right)}^{2}+{\left(\beta_{cur}^{avg}-\beta_{ref}^{avg}\right)}^{2}}$$

Similarly, the average coordinates $Se_{cur}^{avg}=\left(x_{cur}^{avg},y_{cur}^{avg}\right)$ and $Se_{ref}^{avg}=\left(x_{ref}^{avg},y_{ref}^{avg}\right)$ of the semantic objects in the current frame and the reference keyframe are calculated, respectively, and the Euclidean distance between them is calculated as follows:

(15) $$D_{se}=\sqrt{{\left(x_{cur}^{avg}-x_{ref}^{avg}\right)}^{2}+{\left(y_{cur}^{avg}-y_{ref}^{avg}\right)}^{2}}$$

On this basis, $D_{fp}$ and $D_{se}$ are comprehensively measured as follows:

(16) $$D_{sum}=\rho D_{fp}+\left(1-\rho \right)D_{se}$$

If $D_{sum}$ is greater than a set threshold $D_{th}$, the camera’s motion magnitude is is considered too large, and those frames greater than the threshold need to be inserted as keyframes. Otherwise, the frame with the highest $D_{sum}$ is insert as the keyframe, and ρ=0.5 in this paper. Notably, assuming the number of semantic nodes in the current frame and the reference keyframe are Num and $Num^{\prime }$, respectively, and if the difference between Num and $Num^{\prime }$ exceeds a certain threshold $Num_{th}$, i.e., $\left|Num-Num^{\prime }\right|\geq Num_{th}$, a keyframe needs to be inserted in time.

### 3.3. Loop Closure Detection with Combinatorial Graph Entropy

For the traditional bag of words (BoW)-based loop closure detection, the occlusion of dynamic objects will cause changes in the BoW information of the image, resulting in the omission of the true closed-loop frame due to the insufficient number of common words between it and the current frame when screening candidate closed-loop frames, and thus it is unable to successfully detect closed-loops. Therefore, this paper develops a loop closure detection strategy based on combinatorial graph entropy to compensate for the BoW model, effectively suppressing the interference of dynamic changes in the scene on the selection of closed-loop candidate frames. This method quantifies the distribution of feature points in an image by calculating graph entropy, thereby achieving a comparison of similarity between two images. To complete the calculation of graph entropy, we first present a graph construction strategy based on the Euclidean distance between feature points for the graph entropy indicator. Then, to ensure the real-time performance, before calculating the closed-loop similarity, we utilize the semantic information to pre-judge the similarity, and we determine whether to use only the unweighted graph or both the unweighted and weighted graphs for the graph entropy similarity calculation based on the pre-judgment results.

#### 3.3.1. Graph Construction

**Figure 5:**
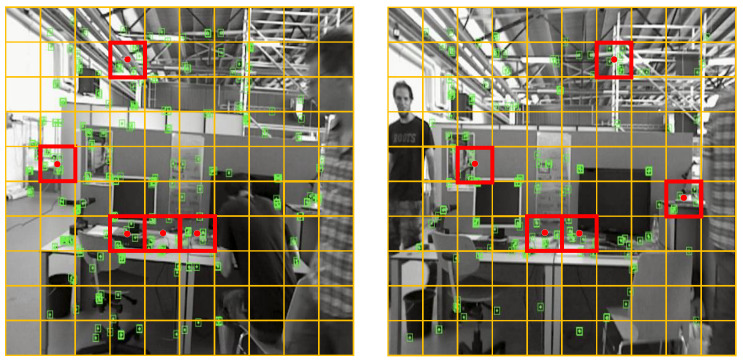
Selection of characterization points.

**Figure 6:**
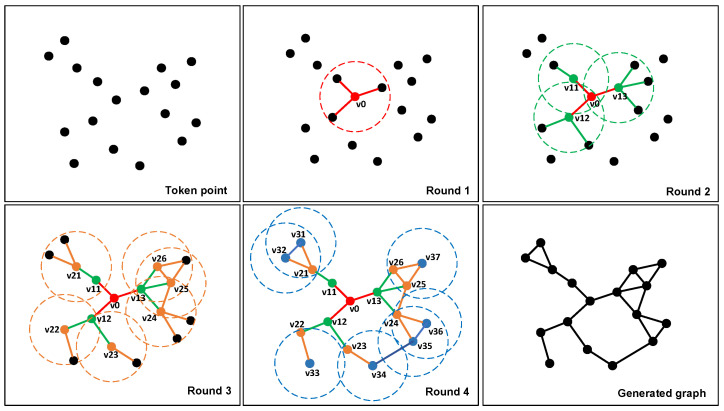
The process of graph construction. The edges constructed in each round are distinguished by different colors, and the circular regions and nodes of different colors indicate the range of each central node and its search radius in each round of the construction process, respectively. The connecting lines, central nodes, and search radius ranges for the first to fourth rounds are represented in red, green, orange, and blue, respectively, while the degree of a node is the number of edges connected to it.

To calculate the graph entropy, it is necessary to develop a comprehensive design strategy of the graph structure. Thus, we propose the following approach:

1. Similarity pre-judgment
  Firstly, we define a semantic vector containing *n* components for the current frame $F_{cur}$ and the candidate frame $F_{cur}$, respectively, i.e.,
  (17) $$X_{cur}=\left(x_{cur}^{1},x_{cur}^{2},x_{cur}^{3},\ldots ,x_{cur}^{n}\right)$$
  (18) $$X_{cand}=\left(x_{cand}^{1},x_{cand}^{2},x_{cand}^{3},\ldots ,x_{cand}^{n}\right)$$
  where $x_{cur}^{i}$ and $x_{cand}^{i}$ denote the number of occurrences of the *i*-th semantic entity in the current frame and candidate frames, respectively. Then, we calculate the cosine similarity between the vectors $X_{cur}$ and $X_{cur}$, i.e.,
  (19) $$S_{cos}^{x}\left(X_{cur},X_{cand}\right)=\frac{X_{cur}\cdot X_{cand}}{∥X_{cur}∥\cdot ∥X_{cand}∥}=\frac{\sum_{i=1}^{n}\left(x_{cur}^{i}\cdot x_{cand}^{i}\right)}{\sqrt{\sum_{i=1}^{n}{\left(x_{cur}^{i}\right)}^{2}}\cdot \sqrt{\sum_{i=1}^{n}{\left(x_{cand}^{i}\right)}^{2}}}$$
  According to the calculation result of Equation (19), an $S_{cos}^{x}$ closer to 1 indicates that the semantic vectors in the two frames are more similar; in this case, using only the unweighted graph can complete the correct judgment. If $S_{cos}^{x}$ is less than a similarity threshold $S_{th}^{x}$, it indicates that the static background of the two frames has undergone significant changes, but it cannot be determined whether they are the same scene or not. In this case, it is necessary to combine the unweighted image and the weighted image to calculate the similarity.
2. Representative point determination
  Typically, when dynamic objects appear in the field of view (FOV), the distribution of feature points in the extracted scene image is undoubtedly disturbed. Hence, before performing similarity calculation, it is necessary to combine the dynamic masks in the current frame and candidate frames to screen out all stable feature points in the two images. More specifically, assuming $Fp_{cur}^{i}$ is any feature point in the current frame with a pixel coordinate of $\left(px_{cur}^{i},py_{cur}^{i}\right)$, if the dynamic masks of both the current and candidate frames do not contain this feature point, then $Fp_{cur}^{i}$ is referred to as a stability feature point.
  After selecting all the stability feature points, we uniformly divide $F_{cur}$ and $F_{cand}$ into rectangular regions and set the size of the length and width of each region to one-tenth of the length and width of the image. Then, we count the number $N_{st}$ of stability feature points within each patch. If $N_{st}$ is greater than the threshold $N_{th}$, the feature points in the region are considered too dense, and the center point of the patch is selected as the “representation point”. When performing the graph structure construction, the characterization points are used as nodes of the graph, while the feature points within their respective patches do not participate in the construction of the graph structure, but feature points within other non-dense patches need to participate as nodes in the construction of the graph structure. As shown in Figure 5, since the number of feature points in the red box is greater than the set threshold, they are considered as dense blocks, and the center coordinates of these blocks are recorded as the coordinates of the representation points.
3. Graph structure generation
  A complete graph structure consists of nodes and edges, and in the case of a weighted graph, the weights of the paths need to be set. To accurately represent the similarity between two images, the connection strategy between nodes needs to ensure that the same scene is not affected by the observation perspective. Otherwise, the same image may have different constructed graph structures due to different observation perspectives. In our case, representation points, feature points in non-dense regions, and the centers of semantic entities (i.e., semantic nodes) together form the nodes of the graph.
  To connect the nodes to form a graph structure, we first set a “flag” for each node, with flag=0 at the initial moment, and set the search radius *R*. Then, the node closest to the center of the image is taken as the starting node $v_{0}$, and $v_{0}$ is connected to the nodes within the search radius *R*, with $v_{0}$ as the center of the connection; that is, let the coordinates of $v_{0}$ be (a,b), and the coordinates of any node $v_{i}$ in the image be $\left(a_{i},b_{i}\right)$. If the following relationship is satisfied, a connection is established $\bar{v_{0}v_{i}}$.
  (20) $$\sqrt{{\left(a-a_{i}\right)}^{2}+{\left(b-b_{i}\right)}^{2}}\leq R$$
  For the $n^{th}$ search, if no other point is searched within *R*, that is, no connection is successfully established, the search radius of the node is expanded to $R^{\prime }$, as follows, until a connection is established.
  (21) $$R^{\prime }=2\left(n-1\right)R$$
  In this way, the first round of connections can be completed.
  The second round of concatenation is sequentially centered on the node that was concatenated in the first round and is searched and concatenated according to the above strategy. Herein, to prevent the graph structure constructed in the case of too sparse keypoints from being too simple, we do not set a threshold for the search radius during the first two rounds of connection.
  Starting from the third round of connection, each round of the connection process takes the node that was connected in the previous round as the center of connection, and any two points are connected only once. Furthermore, to prevent the search radius from being too large and causing too many connections at the edges, the search radius is restricted to expand up to only 3R from the third round onward.
  After completing the graph construction, we further determine whether there are any points in the graph that are not involved in the graph construction and, if so, find the nodes in the constructed graph that have the shortest Euclidean distances from these points and connect them. It should be noted that, when a node is successfully connected, it is necessary to determine whether its flag is equal to 0. If flag=0, the node will be used as the center of the connection for the next search process, and its flag will be set to 1. Figure 6 demonstrates a complete connection process.

According to the above steps, we can construct a complete graph structure $G_{cur}=\left(V_{cur},E_{cur}\right)$ and $G_{cand}=\left(V_{cand},E_{cand}\right)$ based on feature points, semantic nodes, and representation points in $F_{cur}$ and $F_{cand}$, respectively. To calculate the similarity between two graphs, we quantify the structural information of the graphs using the graph entropy $I_{f}\left(G\right)$ [43] and then use the information indices, also known as the degree–degree association indices $I_{f_{exp}^{\Delta }}^{\lambda }\left(G\right)$, to measure the information distance between the maximum graph entropy value $\log\left|V\right|$ and the graph entropy $I_{f}\left(G\right)$ [43,44]. Hence, we can calculate the information indices $G_{cur}$ and $G_{cand}$ for $I_{f_{exp}^{\Delta }}^{\lambda }\left(G_{cur}\right)$ and $I_{f_{exp}^{\Delta }}^{\lambda }\left(G_{cand}\right)$, respectively, and incorporate the value of graph entropy into the similarity calculation to obtain the similarity score between the two graphs.

#### 3.3.2. Graph Entropy Calculation

To meet the application requirements of different scenes and ensure the real-time performance of the algorithm, when the similarity prediction results indicate that the scene structure changes little, we use the graph entropy of unweighted graphs for similarity calculation. When the scene structure changes significantly, it is necessary to combine the graph entropy of weighted and unweighted graphs for a comprehensive evaluation of similarity. The detailed calculation steps are as follows:

1. When it is necessary to use the graph entropy of an unweighted graph, inspired by the work [44], we construct the degree-degree correlation indices corresponding to the two graph structures $F_{cur}$ and $F_{cand}$, respectively, as follows:
  (22) $$I_{f_{exp}^{\Delta }}^{\lambda }\left(G_{cur}\right)=\lambda \left(\log\left|V_{cur}\right|+\sum_{i=1}^{\left|V_{cur}\right|}\frac{f_{exp}^{\Delta }\left(v_{cur}^{i}\right)}{\sum_{j=1}^{\left|V_{cur}\right|}f_{exp}^{\Delta }\left(v_{cur}^{j}\right)}\log\left(\frac{f_{exp}^{\Delta }\left(v_{cur}^{i}\right)}{\sum_{j=1}^{\left|V_{cur}\right|}f_{exp}^{\Delta }\left(v_{cur}^{j}\right)}\right)\right)$$
  (23) $$I_{f_{exp}^{\Delta }}^{\lambda }\left(G_{cand}\right)=\lambda \left(\log\left|V_{cand}\right|+\sum_{i=1}^{\left|V_{cand}\right|}\frac{f_{exp}^{\Delta }\left(v_{cand}^{i}\right)}{\sum_{j=1}^{\left|V_{cand}\right|}f_{exp}^{\Delta }\left(v_{cand}^{j}\right)}\log\left(\frac{f_{exp}^{\Delta }\left(v_{cand}^{i}\right)}{\sum_{j=1}^{\left|V_{cand}\right|}f_{exp}^{\Delta }\left(v_{cand}^{j}\right)}\right)\right)$$
  where λ>0 is a scaling constant; $v_{cur}^{i}$ and $v_{cand}^{i}$ denote a node in $G_{cur}$ and $G_{cand}$, respectively; $\left|V_{cur}\right|$ and $\left|V_{cand}\right|$ denote the number of nodes in $G_{cur}$ and $G_{cand}$, respectively.
  The graph entropy indicator is an information function reflecting the degree-to-degree relationship between nodes. For the graph structure $G_{cur}$ of $F_{cur}$, the calculation of the graph entropy indicator is as follows:
  (24) $$f^{\Delta }\left(v_{cur}^{i}\right)=\alpha^{c_{1}\Delta^{G_{cur}}\left(v_{cur}^{i},1\right)+c_{2}\Delta^{G_{cur}}\left(v_{cur}^{i},2\right)+\cdots +c_{\rho \left(G_{cur}\right)}\Delta^{G_{cur}}\left(v_{cur}^{i},\rho \left(G_{cur}\right)\right)}$$
  If the following hold, one can get $f^{\Delta }=f_{exp}^{\Delta }$.
  (25) $$c_{1}=\rho \left(G_{cur}\right)e^{0},c_{2}=\rho \left(G_{cur}\right)e^{-1},\ldots ,c_{\rho (G_{cur})}=\rho \left(G_{cur}\right)e^{-\rho (G_{cur})+1}$$
  where $\rho \left(G_{cur}\right)$ denotes the diameter of $G_{cur}$ and the connotation of $\Delta^{G_{cur}}\left(v_{cur}^{i},k\right)$ can be found in [43,44]. The node with the shortest path length *j* from can be represented as
  (26) $$S_{j}\left(v_{cur}^{i}\right)=\left\{v_{cur}^{1j},v_{cur}^{2j},v_{cur}^{3j},\ldots ,v_{cur}^{kj}\right\}$$
  where $v_{cur}^{kj}$ denotes the $k^{th}$ node in $G_{cur}$ with path length *j* to $v_{cur}^{i}$, i.e., j-sphere [43]. The shortest paths from $v_{cur}^{i}$ to the $1^{\mathrm{th}},2^{\mathrm{th}},\cdots ,k^{\mathrm{th}}$ node in $S_{j}\left(v_{cur}^{i}\right)$ can be represented as
  (27) $$\begin{array}{c} P_{j}^{1}\left(v_{cur}^{i}\right)=\left\{v_{cur}^{i},v_{cur}^{11},v_{cur}^{12},,\ldots ,v_{cur}^{1j}\right\}\\ P_{j}^{2}\left(v_{cur}^{i}\right)=\left\{v_{cur}^{i},v_{cur}^{21},v_{cur}^{22},,\ldots ,v_{cur}^{2j}\right\}\\ \vdots \\ P_{j}^{k}\left(v_{cur}^{i}\right)=\left\{v_{cur}^{i},v_{cur}^{k1},v_{cur}^{k2},,\ldots ,v_{cur}^{kj}\right\} \end{array}$$
  It should be noted that $v_{cur}^{i}$ may have multiple shortest paths when retrieving each node in $S_{j}\left(v_{cur}^{i}\right)$. In this case, we choose the case where $v_{cur}^{i}$ has the shortest total path after connecting with all the nodes in $S_{j}\left(v_{cur}^{i}\right)$ [43]. $\Delta^{G_{cur}}\left(v_{cur}^{i},k\right)$ is the sum of the differences in degrees between adjacent elements in set $P_{j}^{1}\left(v_{cur}^{i}\right),P_{j}^{2}\left(v_{cur}^{i}\right),\ldots P_{j}^{k}\left(v_{cur}^{i}\right)$, i.e.,
  (28) $$\begin{array}{c} \Delta^{G_{cur}}\left(v_{cur}^{i},k\right)=\left|\delta \left(v_{cur}^{i}\right)-\delta \left(v_{cur}^{11}\right)\right|+\left|\delta \left(v_{cur}^{11}\right)-\delta \left(v_{cur}^{12}\right)\right|+\cdots +\left|\delta \left(v_{cur}^{1(j-1)}\right)-\delta \left(v_{cur}^{1j}\right)\right|\\ +\cdots +\left|\delta \left(v_{cur}^{i}\right)-\delta \left(v_{cur}^{k1}\right)\right|+\left|\delta \left(v_{cur}^{k1}\right)-\delta \left(v_{cur}^{k2}\right)\right|+\cdots +\left|\delta \left(v_{cur}^{k(j-1)}\right)-\delta \left(v_{cur}^{kj}\right)\right| \end{array}$$
  where σ denotes the degree of the node.
2. When it is necessary to use the graph entropy of a weighted graph, assuming that the weighted graphs generated by $F_{cur}$ and $F_{cand}$ are as follows:
  (29) $$G_{cur}=\left(V_{cur}^{1:i},E_{cur}^{1:j},W_{cur}^{1:j}\right)$$
  (30) $$G_{cand}=\left(V_{cand}^{1:m},E_{cand}^{1:n},W_{cand}^{1:n}\right)$$
  where the weights $W_{cur}^{1:j}$ and $W_{cand}^{1:n}$ denote the set of Euclidean distances between the two endpoints corresponding to all the paths in $F_{cur}$ and $F_{cand}$, respectively. In this case, we use Shannon entropy to calculate the graph entropy of the two graphs [45]. Herein, the Shannon entropies of $G_{cur}$ and $G_{cand}$ are defined as follows:
  (31) $$I\left(G_{cur},W_{cur}^{1:j}\right)=-\sum_{l_{cur}^{s}\in E_{cur}^{1:j}}p_{l_{cur}^{s}}\log\left(p_{l_{cur}^{s}}\right)$$
  (32) $$p_{l_{cur}^{s}}=\frac{W_{cur}^{1:j}\left(l_{cur}^{s}\right)}{\sum_{l_{cur}^{s}\in E_{cur}^{1:j}}W_{cur}^{1:j}\left(l_{cur}^{s}\right)}$$
  (33) $$I\left(G_{cand},W_{cand}^{1:n}\right)=-\sum_{l_{cand}^{t}\in E_{cand}^{1:n}}p_{l_{cand}^{t}}\log\left(p_{l_{cand}^{t}}\right)$$
  (34) $$p_{l_{cand}^{t}}=\frac{W_{cand}^{1:n}\left(l_{cand}^{t}\right)}{\sum_{l_{cand}^{t}\in E_{cand}^{1:n}}W_{cand}^{1:n}\left(l_{cand}^{t}\right)}$$
  where $l_{cur}^{s}\in E_{cur}^{1:j}$, $l_{cand}^{t}\in E_{cand}^{1:n}$ denote the $s^{th}$ edge of $G_{cur}$ and the $t^{th}$ edge of $G_{cand}$, respectively.
  Compared with the unweighted graph, the weighted graph introduces the distance between the nodes as the weights of the paths when performing the similarity calculation, which can reflect the change in the Euclidean distance between the nodes due to the change in depth to a certain extent. Therefore, the weighted graph is more suitable for loop closure detection in the case of large changes in scene structure.

#### 3.3.3. Similarity Calculation

After obtaining the graph entropy of $F_{cur}$ and $F_{cand}$, loop closure detection can be performed by measuring the similarity between the graph entropies. Dehmer et al. [46] proposed four similarity measurement criteria, and through experimental verification, we found that when using graph entropy $I_{f_{exp}^{\Delta }}^{\lambda }\left(G\right)$ for closed-loop similarity calculation, since there are a large number of feature points extracted from each image, the calculated graph entropy value for each image is much greater than 1. This may result in negative similarity scores calculated by three of the similarity metrics, which does not conform to the definition of the similarity metrics. Thus, in terms of the first measurement criterion, we constructed the similarity calculation approach for $F_{cur}$ and $F_{cand}$ as follows:

(35) $$S_{ge}\left(I\left(G_{cur}\right),I\left(G_{cand}\right)\right)=\frac{1}{1+{\left|I\left(G_{cur}\right)-I\left(G_{cand}\right)\right|}^{\alpha }}$$

where α>1 is an arbitrary positive integer.

1. When only unweighted graphs need to be used, the graph entropies of $G_{cur}$ and $G_{cand}$ are the corresponding degree–degree correlation indices, respectively, i.e.,
  (36) $$I\left(G_{cur}\right)=I_{f_{exp}^{\Delta }}^{\lambda }\left(G_{cur}\right),I\left(G_{cand}\right)=I_{f_{exp}^{\Delta }}^{\lambda }\left(G_{cand}\right)$$
  Hence, the final graph entropy similarity score is as follows:
  (37) $$S_{sum}=S_{ge}\left(I_{f_{exp}^{\Delta }}^{\lambda }\left(G_{cur}\right),I_{f_{exp}^{\Delta }}^{\lambda }\left(G_{cand}\right)\right)$$
2. When it is necessary to use both unweighted and weighted graphs, the similarity scores of the corresponding unweighted graphs of $F_{cur}$ and $F_{cand}$ are calculated as follows:
  (38) $$S_{ge}\left(I_{f_{exp}^{\Delta }}^{\lambda }\left(G_{cur}\right),I_{f_{exp}^{\Delta }}^{\lambda }\left(G_{cand}\right)\right)$$
  Subsequently, let the graph entropies of $G_{cur}$ and $G_{cand}$ be the corresponding Shannon entropies, respectively, i.e.,
  (39) $$I\left(G_{cur}\right)=I\left(G_{cur},W_{cur}^{1:j}\right),I\left(G_{cand}\right)=I\left(G_{cand},W_{cand}^{1:n}\right)$$
  The similarity scores of the corresponding weighted graphs of $F_{cur}$ and $F_{cand}$ are calculated as follows:
  (40) $$S_{Wge}\left(I\left(G_{cur},W_{cur}^{1:j}\right),I\left(G_{cand},W_{cand}^{1:n}\right)\right)$$
  Further, the following combinatorial graph entropy similarity score is obtained by evaluating the two similarity scores together:
  (41) $$S_{sum}=\lambda S_{ge}+\left(1-\lambda \right)S_{Wge}$$
  Finally, we utilize the calculated $S_{sum}$ as the graph entropy similarity score for $F_{cur}$ and $F_{cand}$. In this paper, in the case of using both unweighted and weighted graphs, λ=0.5.

**Figure 7:**
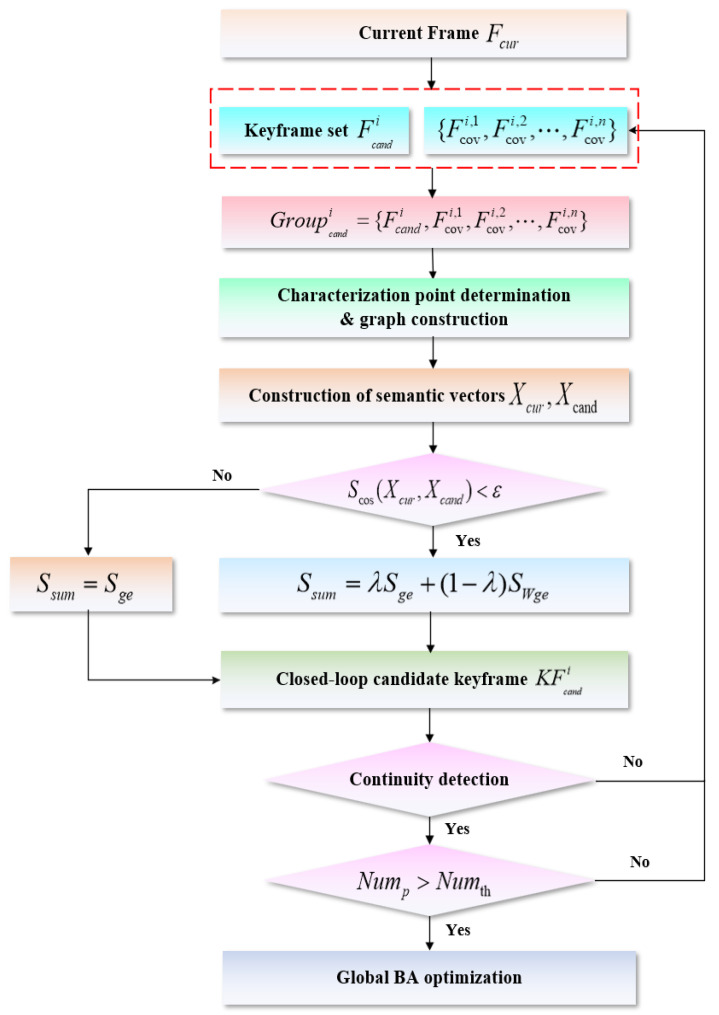
Scenarios for the loop closure detection based on combinatorial graph entropy.

Based on the above analysis, the flow of loop closure detection based on combinatorial graph entropy is shown in Figure 7. Firstly, among the keyframes that are not connected to the current frame $F_{cur}$, a set $\Xi =\{F_{cand}^{i}\}$ of keyframes that satisfy a threshold value for the number of common words with the current frame $F_{cur}$ is selected using the BoW model, and the *n* keyframes in Ξ that have the highest degree of covisibility with $F_{cand}^{i}$ are formed into a candidate frame group $\left\{F_{\mathrm{cov}}^{i,1},F_{\mathrm{cov}}^{i,2},\cdots ,F_{\mathrm{cov}}^{i,n}\right\}$, and $F_{cand}^{i}$ is further included in this candidate frame group, i.e.,

(42) $$Group_{cand}^{i}=\left\{F_{cand}^{i},F_{\mathrm{cov}}^{i,1},F_{\mathrm{cov}}^{i,2},\cdots ,F_{\mathrm{cov}}^{i,n}\right\}$$

Then, according to the characteristics of the scene, the current frame $F_{cur}$ and the candidate frames in each candidate frame group $Group_{cand}^{i}$ are selected one by one to carry out the graph entropy similarity score calculation by the corresponding method, and the total score of each group and the highest score within the group are recorded. Next, the highest-scoring frame within the group is selected as the closed-loop candidate keyframe from the candidate group with the highest total score. If the closed-loop candidate keyframe passes the subsequent continuity detection and the number of matched map points with the current frame exceeds the threshold, i.e., $Num_{p}>Num_{\mathrm{th}}$, the closed-loop candidate keyframe is considered to form a closed-loop with the current frame, in our case, $Num_{th}=40$. In turn, the global bundle adjustment (BA) optimization is performed to eliminate the cumulative error based on the pose relationship between the current frame and the candidate frames.

## 4. Simulation and Experimental Results

The simulation studies and experimental testing with a mobile robot for the proposed algorithm are given in this section. All of the experiments were performed on a laptop with an Intel i5-12500H CPU, 16 GB of RAM, and NVIDIA RTX3050 GPU, running under the Ubuntu 18.04 operating system. More details about the experimental configurations are listed in Table 1.

To validate the performance of the algorithm in dynamic scenes, we selected high-dynamic Walking sequences from the *TUM* dataset created by the Technical University of Munich, Germany, to carry out simulation studies. The *TUM* dataset includes complete RGB-D image sequences and the camera’s true trajectory. In terms of the camera’s motion state, the walking sequence includes the walking_xyz sequence moving along the xyz axis, the walking_halfsphere sequence moving along a hemispherical trajectory, the walking_rpy sequence rotating along yaw and pitch angles, and the walking_static sequence in a stationary state. In this study, we compared and analyzed our algorithm with classic ORB-SLAM2, DynaSLAM, and existing state-of-the-art SLAM algorithms. Furthermore, to evaluate the localization accuracy of the algorithm, we employed absolute trajectory error (ATE) and relative translation error (RTE) as measurement indicators, in which the ATE is the error between the camera’s true trajectory and the predicted trajectory, which intuitively reflects the global positioning accuracy of the algorithm, while RTE indicates the difference between the true pose and predicted pose of the camera at the same time point, used to evaluate the drift of the SLAM system.

### 4.1. Simulation Studies Under the TUM Dataset

Table 2 lists the absolute trajectory error (ATE) of different algorithms on the *TUM* dataset, where “—” indicates that the algorithm did not provide relevant experimental results, while bold indicates the optimum value in the corresponding row. It can be seen that compared to ORB-SLAM2, the absolute trajectory accuracy of our algorithm improved by 97.01% on average under walking high-dynamic sequences. This is due to the fact that ORB-SLAM2 treated all the extracted feature points as static and thus inevitably suffered from the interference of dynamic features in the pose estimation. Instead, we combined the dynamic mask obtained by YOLOv8-seg with the MAD of depth to accurately remove the dynamic feature points, ensuring that the ones participating in the pose estimation are all high-confidence static feature points, effectively improving the accuracy of pose estimation.

**Figure 8:**
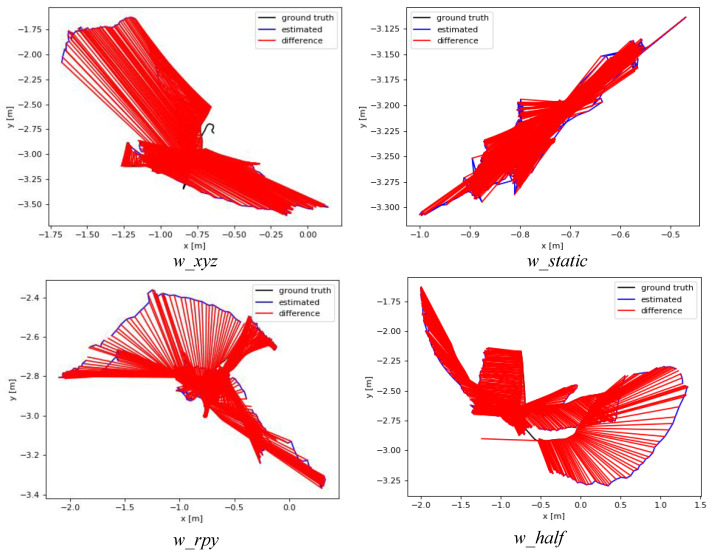
The ATE of ORB-SLAM2 on the walking sequences.

**Figure 9:**
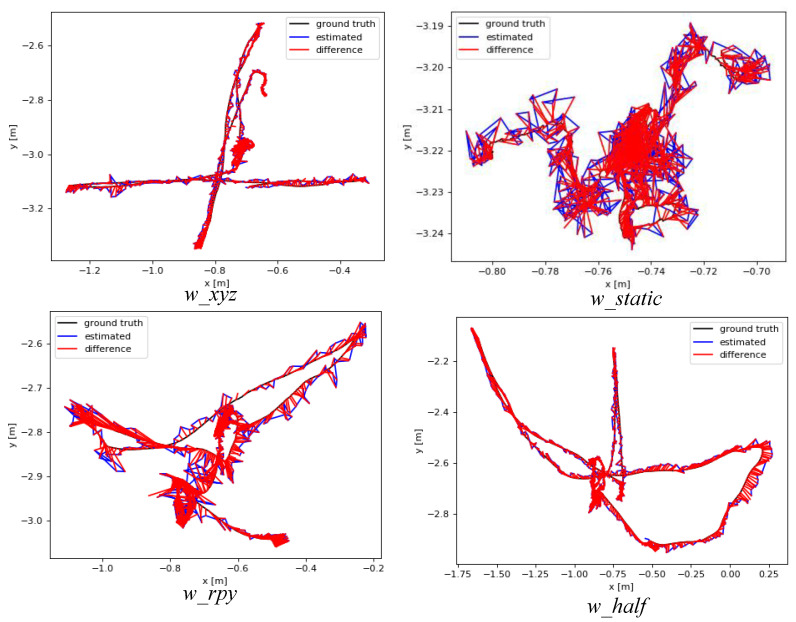
The ATE of our algorithms on the walking sequences.

Figure 8 and Figure 9 illustrate the absolute trajectory error between our algorithm and ORB-SLAM2, where the red area denotes the error between the predicted trajectory and the true trajectory. It can be found that, in high-dynamic walking sequences, ORB-SLAM2 treated feature points on dynamic objects (people) as static and involved in camera’s pose estimation, resulting in significant error accumulation, whereas our algorithm utilized YOLOv8-seg and MAD in the front end to detect and eliminate these dynamic feature points, and the predicted trajectories obtained were better fitted to the real trajectories.

Compared with other tested algorithms, our algorithm also illustrated significant improvement in some scenes. Although the accuracy of our algorithm was slightly lower than the other mentioned algorithms on walking_static sequences, the ATE of our algorithm was minimized on walking_xyz, as well as walking_half and walking_rpy sequences with higher dynamics. Quantitatively, on the walking_rpy sequence, compared with several other algorithms, the absolute trajectory accuracy of our algorithm was improved by 14.75% on average. On walking_half sequences with complex camera movements, our algorithm improved by 5.44%, 5.07%, and 16.20% compared to DynaSLAM, Blitz SLAM [49], and the algorithm proposed by Ji [47], respectively, while improving by 23.10% and 19.80% compared to MOLO-SLAM [50] and the algorithm proposed by Sun [48], respectively. In addition, although this paper mainly focuses on applications in high-dynamic scenes, the proposed algorithm still demonstrated good accuracy in low-dynamic scenes (e.g., the sitting dataset). On the sitting_half and sitting_static sequences, the accuracy of our algorithm was improved by 9.9% and 27% compared with ORB-SLAM2, respectively.

In Table 3, compared with ORB-SLAM2, the RTE of our algorithm reduced the RMSE on high-dynamic sequences by 93.16% on average. This indicates that after obtaining high-confidence static feature points, the relative pose changes calculated by our algorithm were more in line with the real situation. Compared to other tested algorithms except ORB-SLAM2, the proposed algorithm maintained a low RTE even in high-dynamic scenes. On the walking_xyz sequence, the RTE of our algorithm was reduced by 12.38% on average compared with other algorithms. It is worth mentioning that the algorithm in this paper still performs well on the walking half and walking rpy columns, where the camera moves violently. On these two sequences, the RTE of our algorithm is reduced by 33.3% and 15.68%, respectively. Especially on the walking_half sequence, our algorithm performed the best, with a maximum reduction of 44.07% in RTE. On the walking_rpy sequence, compared with DynaSLAM, Blitz SLAM, MOLO-SLAM, Ji’s algorithm, and Sun’s algorithm, our algorithm improved by 1.20%,13.31%, 24.07%, 12.95%, and 26.91%, respectively. It is worth mentioning that although the proposed algorithm focuses on high-dynamic scenes, the RTE in low-dynamic scenes also maintains a similar level to other algorithms.

**Figure 10:**
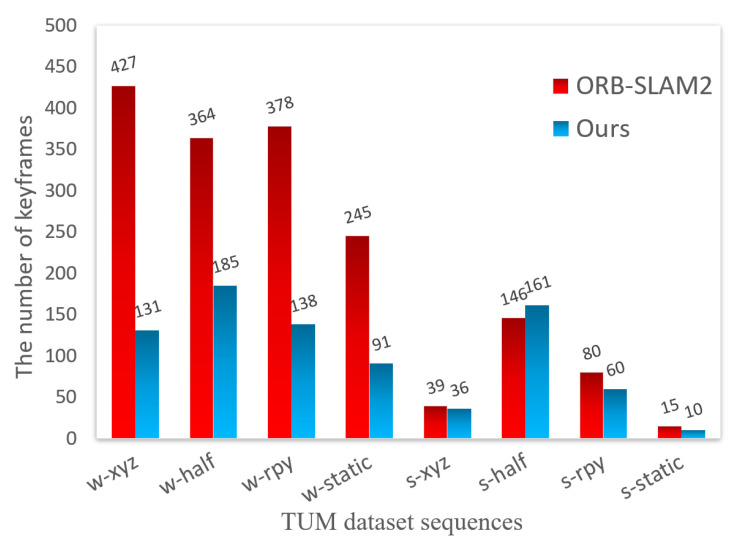
Comparison of the number of keyframes extracted by different algorithms on the *TUM* dataset sequences.

Figure 10 compares the number of keyframes for ORB-SLAM2 and our algorithm under high- and low-dynamic sequences. Since ORB-SLAM2 does not cull dynamic objects, the number of selected keyframes was excessive and contained much redundancy. In contrast, the algorithm in this paper excluded the influence of dynamic objects, so the number of keyframes selected in high-dynamic scenes was significantly reduced. In the four datasets of xyz, halfsphere, rpy, and static in the fr3_walking_sequence, the number of keyframes selected by our algorithm decreased by 69.32%, 49.17%, 63.49%, and 62.85% compared to ORB-SLAM2, respectively. In low-dynamic scenes, the number of keyframes selected by our algorithm was only slightly reduced compared to that of ORB-SLAM2, and even in the halfsphere dataset of the fr3_sitting_ sequence, the number of keyframes selected by the ORB-SLAM2 algorithm was less than that of our algorithm. This is because, when the camera moves along the hemisphere trajectory, the instance segmentation network may fail to segment, which leads to inconsistent feature point removal in the previous and subsequent frames, resulting in a slight increase in the number of keyframe selections. However, compared to ORB-SLAM2, the number of keyframes selected by the proposed algorithm on xyz, rpy, and static sequences was reduced by 7.6%, 25%, and 33%, respectively. Therefore, in most low-dynamic scenes, the keyframe selection strategy of our algorithm still maintains excellent performance.

**Figure 11:**
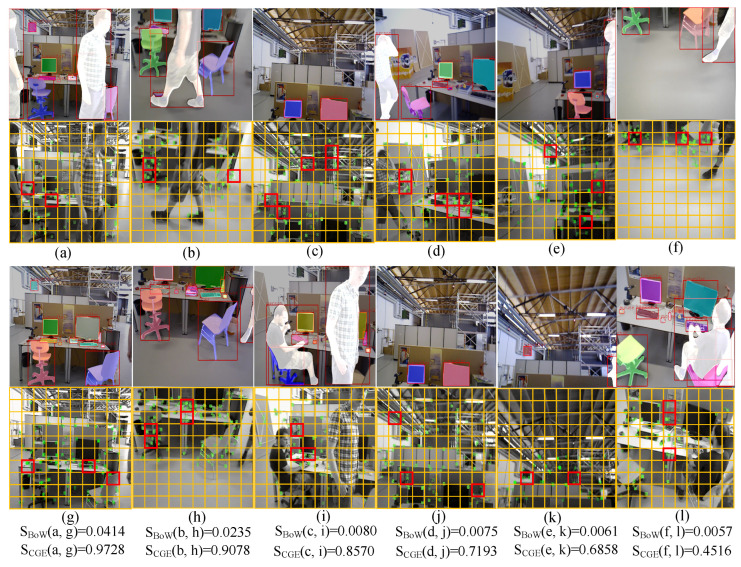
Similarity calculation results for different image pairs on the fr3_walking sequence.

To verify the accuracy and reasonableness of the similarity calculation strategy for loop closure detection in this paper, we selected six sets of discontinuous images from four different datasets of the fr3w_taking sequence and then calculated the BoW similarity SBoW and graph entropy similarity $S_{CGE}$ for each set of images. As shown in Figure 11, despite the interference of the dynamic pedestrians, the camera’s poses in Figure 11a,b were almost the same as those in Figure 11g,h, respectively, and the calculated $S_{CGE}$ exceeded 0.9. In contrast, the camera’s poses in Figure 11c,d were slightly different from those in Figure 11i,j, respectively, resulting in a decrease in $S_{CGE}$ and $S_{BoW}$ compared to the previous two sets of images. In addition, the camera’s poses in Figure 11e,f differed the most from those in Figure 11k,l, respectively, among the six sets of images. Thus, the calculated $S_{BoW}$ and $S_{CGE}$ were also the lowest. We could reasonably conclude that the more similar the two scene images are, the higher the calculated image entropy similarity score, and the combinatorial graph entropy-based similarity calculation strategy can serve as a constraint condition to compensate for the similarity of BoW.

To verify the impact of different modules on ATE in the proposed algorithm, we conducted ablation experiments on the f3_walking sequence. In the simulation, we still employed RMSE to measure the deviation between the estimated pose and the true pose and further adopted standard deviation (SD) to reveal the overall dispersion between them. If both RMSE and SD are relatively small, it indicates that the estimated pose of the algorithm is closer to the true pose. In Table 4, Ours (Y) denotes that the algorithm only used Yolov8-seg to remove dynamic feature points. In this case, compared with ORB-SLAM2, the RMSE and SD of the ATE of the proposed algorithm on high-dynamic sequences were reduced by 96.4% and 95.79% on average, respectively. From this, it can be known that combining the dynamic mask provided by Yolov8-seg to eliminate the interference of dynamic factors can greatly improve the accuracy of the algorithm in dynamic environments. Ours (Y + M) denotes the combination of Yolov8-seg and depth’s MAD in the algorithm for dynamic feature point elimination. Clearly, the accuracy of the algorithm was improved compared to Ours (Y) due to the accurate elimination of dynamic points missed at the edges of dynamic objects by Ours (Y + M), especially on the w_rpy dataset, where the RMSE and SD of the algorithm’s ATE were reduced by 17.6% and 36.9%, respectively. This is because the images extracted when the camera’s motion is relatively intense tend to be blurry, which can cause deviation in the segmentation results of the network. However, the MAD of depth can compensate for the accuracy of the algorithm well. Ours (Y + M + K) denotes the further incorporation of the keyframe optimization strategy based on the first two improvements. It can be observed that the algorithm improved the quality of keyframes and reduced the error accumulation caused by redundant keyframes, further reducing the ATE of the algorithm. Especially in the w_static dataset, the RMSE and SD of the ATE of Ours (Y + M + K) compared to Ours (Y + M) were reduced by 7.92% and 5.8%, respectively.

Table 5 compares the average tracking time and detection/segmentation time of our algorithm with some mainstream SLAM algorithms for each frame. Since the proposed algorithm utilized the instance segmentation network YOLOv8-seg to eliminate dynamic objects at the front end, the tracking time was slightly higher than that of ORB-SLAM2, but the tracking of our method was made more stable by eliminating the interference of dynamic features. In terms of time consumption for segmentation and tracking, our algorithm decreased by 97.05% and 91.02% compared to Dyna-SLAM using Mask R-CNN and by 96.36% and 95.89% compared to YOLO-SLAM using YOLOv3, respectively. This is mainly due to the fact that we exploited the more lightweight YOLOv8-seg model and optimized the detection results using the MAD of depth, which is more concise and efficient. Compared with DO-SLAM using YOLOv5, the proposed algorithm reduced the time consumption by 68.95% and 76.74%, respectively, which is because YOLOv8 runs faster on GPUs than YOLOv5. In addition, since YOLO-SLAM utilized a target detection network, it required more computational resources to achieve accurate elimination of dynamic feature points, whereas we adopted an instance segmentation network, which is less computationally intensive when performing the compensation and thus the proposed method had better real-time performance.

**Figure 12:**
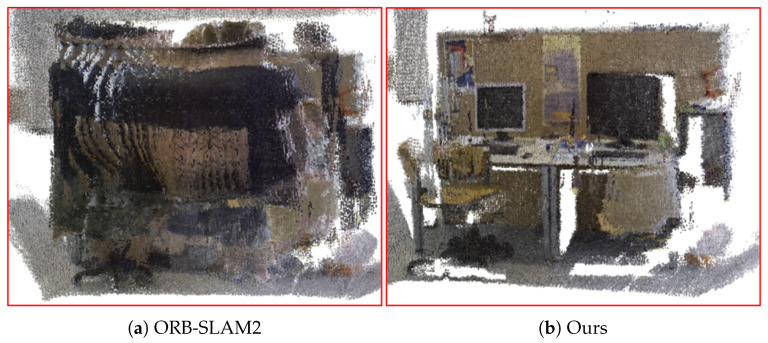
The semantic octree mapping effects of different algorithms on the fr3 walking_static sequence.

Figure 12 shows the mapping effects of different algorithms on the fr3 walking_static sequence. Since ORB-SLAM2 does not involve the processing of dynamic objects, pedestrians were also included as part of the background in the mapping process, resulting in the generated map being misaligned and cluttered. In contrast, our algorithm detected and eliminated dynamic feature points in the front end, effectively suppressing the interference of dynamic objects. Hence, the constructed semantic octree map exhibited good consistency and clarity and can accurately reflect the real scene.

To quantitatively assess the quality of semantic mapping, we utilize semantic integrity and semantic fluctuation proposed by Zhang et al. [51] as metrics. The higher the semantic integrity, the more complete the semantic information of the semantic map. The lower the fluctuation, the more stable the semantic information of the semantic map. Assuming that $T_{O}$ is the number of squares used to represent the color of a certain entity, $T_{N}$ is the number of squares within the cube approximating that semantic entity, and the number of keyframes involved in mapping is N, the integrity score in each keyframe is as follows:

(43) $$S_{int}\left(N\right)=\frac{T_{O}}{T_{N}}$$

In this way, the calculation for global semantic integrity and global semantic fluctuation are, respectively, as follows

(44) $$Score_{INT}=\sum_{N}\left[\left(\frac{T_{N}}{\sum_{N}*T_{N}}\right)*S_{int}\left(N\right)\right]$$

(45) $$Score_{flu}=\frac{\sum_{N}\left\{T_{N}*{\left[S_{int}\left(N\right)-Score_{INT}\right]}^{2}\right\}}{\sum_{N}T_{N}}$$

**Figure 13:**
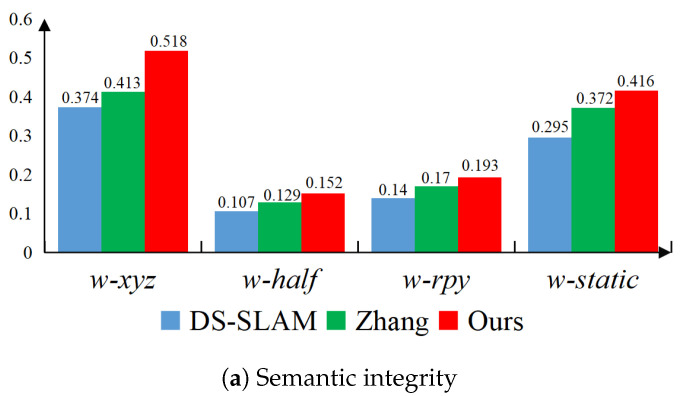

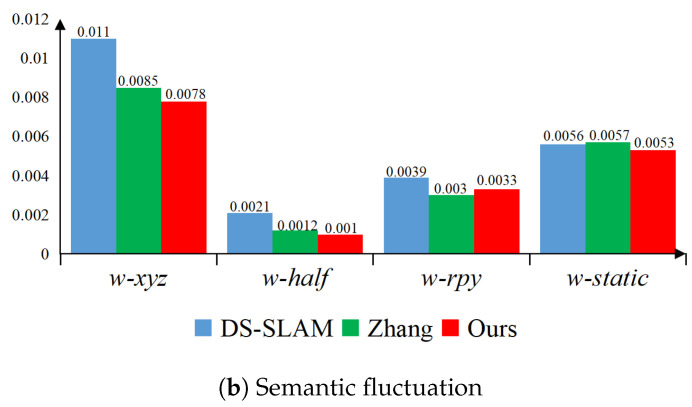
Comparison of semantic integrity and fluctuation of different algorithms under the Tum dataset.

Figure 13 compares the semantic integrity and semantic fluctuation scores of DS-SLAM [52], Zhang et al. [51], and our algorithm on different sequences. Apparently, compared to the SegNet network used by DS-SLAM and Zhang et al. [51], this work utilized the YOLOv8-seg instance segmentation network with higher accuracy, resulting in a semantic octree map with higher integrity and less fluctuation.

### 4.2. Experimental Testing with a Mobile Robot

**Figure 14:**
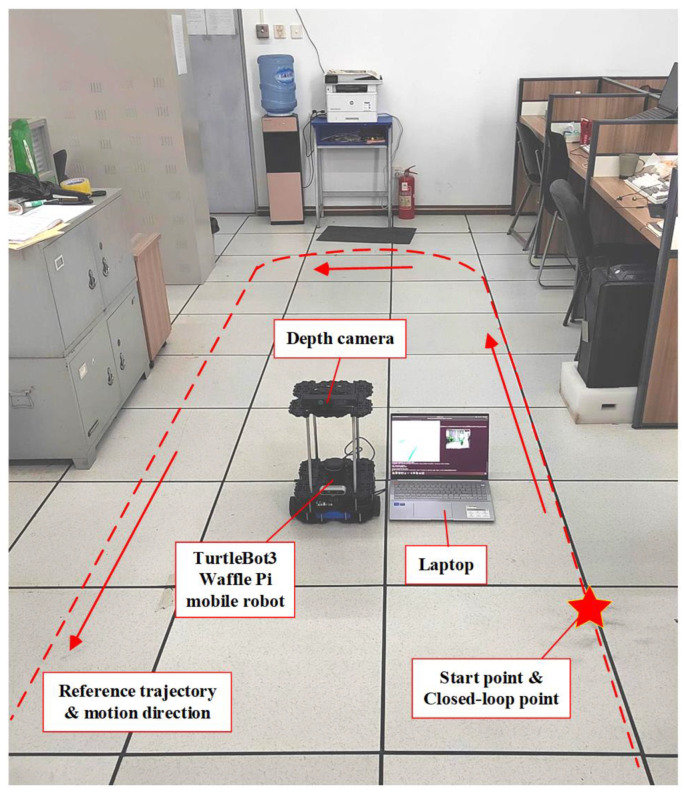
Mobile robot and experimental scene.

To verify the feasibility and effectiveness of the proposed algorithm in real scenes, we conducted experimental tests using a TurtleBot3 Waffle Pi (ROBOTIS, Seoul, Republic of Korea) mobile robot equipped with an Intel^®^ RealSense™ D435i depth camera in a laboratory with an area of 8.4 m × 6.4 m, as shown in Figure 14. In the experiment, the mobile robot moves along the red dashed line in the direction of the arrow. Meanwhile, a pedestrian walked back and forth within the robot’s FoV at a speed of 0.5 m/s to simulate a dynamic scene.

**Figure 15:**
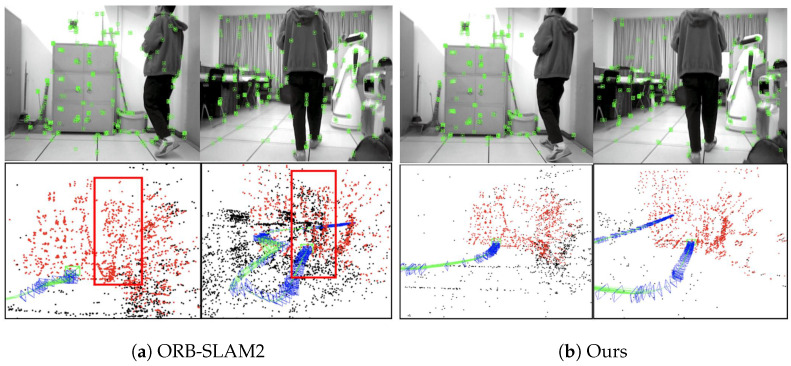
The running effects of different algorithms in a real scene. The top row shows the feature point extraction results, while the bottom row represents the corresponding 3D sparse point cloud map.

Figure 15 demonstrates the running effects of our algorithm and ORB-SLAM2 in a real scene. From Figure 15a, it can be seen that ORB-SLAM2 extracted many dynamic feature points on pedestrians, resulting in many cluttered point clouds (the part contained in the red box) in the generated 3D sparse maps; whereas, in Figure 15b, our algorithm accurately detected the dynamic feature points in the scene and removed them, which effectively eliminated the interference of the dynamic targets, and thus the sparse point cloud map constructed by our algorithm was more consistent with the actual scene.

**Figure 16:**
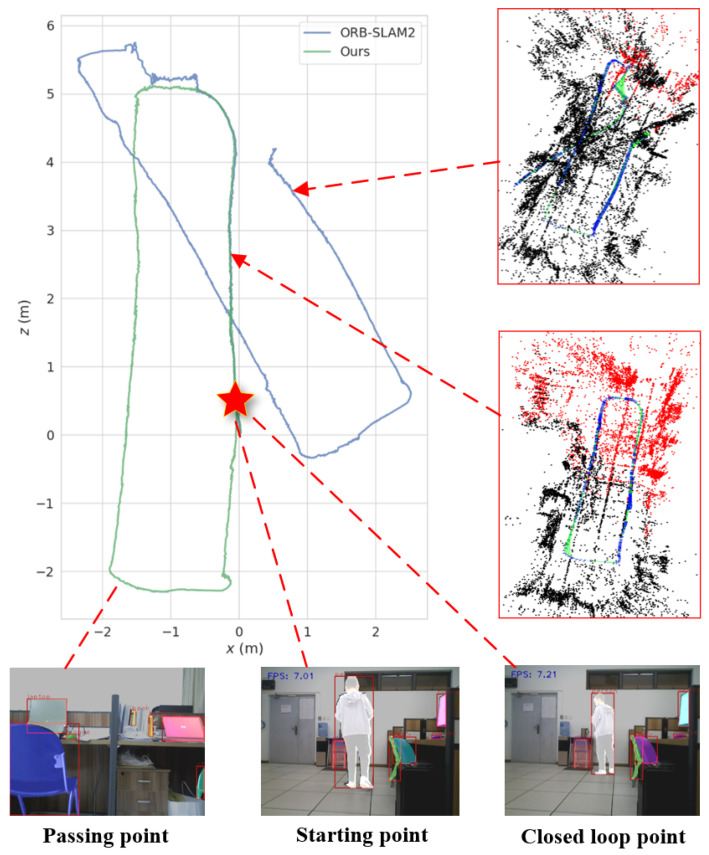
Trajectory estimation results of different algorithms in a real scene.

Figure 16 further illustrates the global trajectory estimation results of ORB-SLAM2 and the proposed algorithm in real scenes. Clearly, since ORB-SLAM2 introduced a large amount of dynamic feature points into the robot’s pose estimation, the performance of loop closure detection in ORB-SLAM2 had significantly decreased, making it difficult to perform BA optimization in a timely manner, and thus, there was a serious deviation between the estimated trajectory and the reference trajectory. In contrast, the proposed algorithm combined YOLOv8-seg with the MAD of depth for dynamic feature point elimination in the front-end, which ensured that the static feature points involved in pose estimation had high confidence. Moreover, with the elimination of dynamic interference, the proposed algorithm was able to accurately detect closed loops to correct the robot’s pose, so the predicted trajectory was more in line with the reference trajectory.

To verify the performance of the loop closure detection in real scenes, we employed “precision” and “recall” as evaluation metrics, in which the precision refers to the proportion of real closed-loops detected by the algorithm among all closed loops, while recall is the percentage of actual closed-loop times detected by the algorithm among all loop times in the scene. If positive indicates that a closed loop is detected and negative indicates that no closed loop is detected, then the precision “P” and recall “R” are, respectively, represented as follows:

(46) $$P=\frac{TP}{TP+FP},R=\frac{TP}{TP+FN}$$

where TP denotes true positive, FP denotes false positive, and FN denotes false negative.

**Figure 17:**
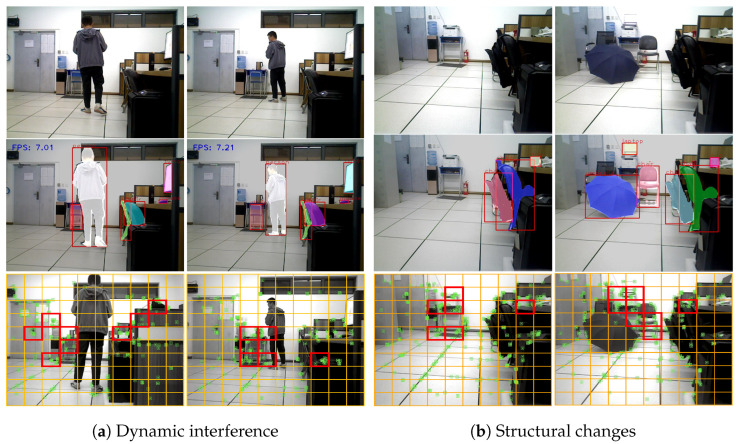
Detection results of closed-loop regions in different scenes. The top row is the input image, the middle row is the instance segmentation results, and the bottom row is the characterization point selection results.

In this study, we carried out loop closure detection experiments for scenes with dynamic disturbances and changes in structure, respectively. In Figure 17a, the left and right denote the starting point and closed-loop point of the robot, respectively, and a dynamic disturbance was simulated by a randomly moving pedestrian within the robot’s FoV. In Figure 17b, the left and right represent the starting point and closed-loop point of the robot, respectively. At the closed-loop point, we added two chairs, a laptop, and an umbrella to simulate the scene of structural changes. In the experiment, the robot ran a total of eight circles along the reference trajectory, with each circle containing a closed loop. The experimental results are shown in Table 6.

In Figure 17a, when the right image was selected as a keyframe, the system performed loop closure detection. From the segmentation results, it can be seen that the static objects detected in both images were three chairs, one mouse, and one monitor. Therefore, the semantic vectors constructed based on static semantic entities for both images were (2,1,1), and the cosine similarity was equal to 1, indicating that the two images belong to general dynamic scenes. In this case, the unweighted image was selected for graph entropy similarity calculation. Then, the BoW model was used to preliminarily screen candidate frames for the existing keyframe groups, and the selected candidate frame groups were further validated using the proposed algorithm. The final BoW similarity score for the two frames was calculated to be 0.0542, and the graph entropy similarity score was 0.9004. We find that both algorithms obtained high similarity scores, so among the existing keyframe groups, the left image was finally selected as a closed-loop candidate keyframe and finally passed the continuity judgment and the detection of the number of matched map points, which will be subsequently used as a closed-loop keyframe to involve in the pose optimization. From Table 6, we observe that there were two false negatives (i.e., FN = 2) when using only BoW for loop closure detection, indicating that BoW did not detect closed loops in the loop area in a timely manner. This is because dynamic object occlusion reduced the BoW similarity between images, leading to loop closure detection failure. However, our algorithm can effectively eliminate the interference of dynamic objects and had an advantage in recall in the same scene. It is worth mentioning that both loop closure detection algorithms demonstrated high accuracy without false positives (FP = 0), i.e., without misjudging non loop areas as loop points.

Likewise, in Figure 17b, we calculated the cosine similarity between the two frames to be 89.44%, indicating that the static background structure in the images has changed, and a weighted image needs to be introduced. By calculation, the BoW similarity score was 0.00398, while the similarity scores for unweighted and weighted graph entropy were 0.9053 and 0.925, respectively, which in turn leads to the combined graph entropy score of 0.9152. Herein, the left image also passed the screening of closed-loop candidate keyframes and underwent continuity detection and the detection of the number of matched map points. Therefore, it is used as a loop frame to participate in subsequent pose optimization.

From Table 6, it can be found that changes in the static background structure result in changes in the BoW information of the image. Since the closed-loop region was the only the static structure that changed without the interference of dynamic objects, the loop closure detection using the BoW model was not significantly affected, and only one false negative occurred, while the algorithm in this paper did not have false negatives and still demonstrated a high recall. Furthermore, in a real scene, the average tracking time per frame of our algorithm was 28.752 ms, while the average segmentation time was 23.104 ms, so it exhibits good real-time performance.

**Figure 18:**
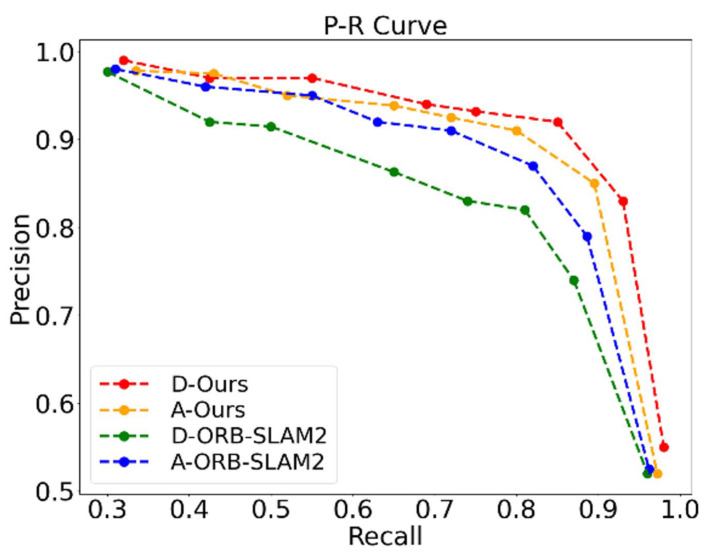
“P-R” curves of different loop closure detection methods.

Figure 18 further illustrates the “P-R” curves of our algorithm and ORB-SLAM2 in the dynamic scene and the scene where the static background structure changes, respectively. Among them, “D-Ours” and “D-ORB-SLAM2” represent the “P-R” curves of the two algorithms in the dynamic scene.

Since ORB-SLAM2 cannot eliminate the influence of dynamic objects in the scene, it may result in a significant deviation between the calculated BoW similarity score and the actual one, whereas our algorithm eliminated the interference of dynamic feature points by using semantic information in the front end and maximized the retention of static feature points with high confidence. Meanwhile, before calculating the graph entropy similarity, the proposed algorithm first drew on the BoW model for preliminary judgment of candidate frames and then combined the BoW score and the graph entropy similarity score for the selection of closed-loop candidate keyframes to compensate for the BoW score. In addition, to prevent the vacant region in the image after removing dynamic objects from affecting the distribution of feature points in the image, our algorithm first determined the representation points of two frames of images. Therefore, our algorithm exhibited higher accuracy compared to ORB-SLAM2 with the same recall in dynamic scenes. When the static structure in the scene was changed, the “P-R” curves of our algorithm and ORB-SLAM2 were shown as “A-Ours” and “A-ORB-SLAM2” in Figure 18, respectively. Since ORB-SLAM2 only used the BoW model for similarity calculation, although the interference of dynamic targets in the scene was relatively small, changes in the static background structure caused changes in the BoW score, which had a negative impact on the accuracy of loop closure detection. In this scene, the proposed loop closure detection algorithm used a combinatorial graph entropy score to compensate for the BoW score, which avoided the effect of static structure changes to a certain extent, and thus the accuracy of the algorithm was slightly improved compared to ORB-SLAM2.

**Figure 19:**
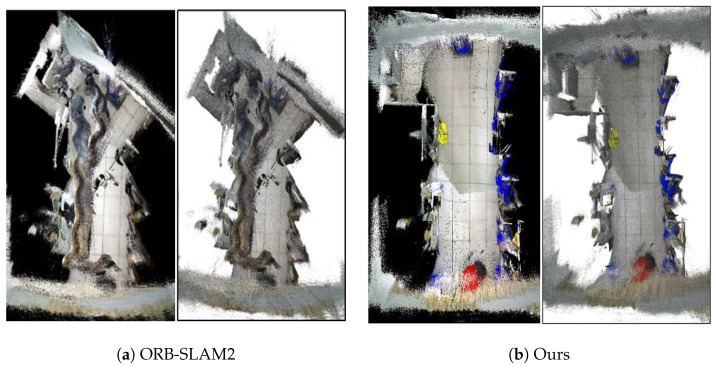
Comparison of global maps of different algorithms in a real scene. In (**a**,**b**), the left is a 3D dense semantic map, and the right is the corresponding global semantic octree map.

To validate the global mapping effect of the algorithm in dynamic scenes, we constructed a 3D dense semantic map and the corresponding 3D semantic octree map of the real dynamic scene shown in Figure 19 using ORB-SLAM2 and the proposed algorithm, respectively. We can reasonably conclude from Figure 19 that, since ORB-SLAM2 failed to eliminate the interference of dynamic target, a large amount of feature points on the pedestrian were involved in feature matching and robot pose estimation, resulting in the constructed map not only being distorted but also the pedestrian’s motion trajectory appearing in the map. In contrast, the proposed algorithm effectively eliminated dynamic objects in the front end and obtained more accurate pose estimation by using static features with high confidence, so the constructed octree maps were more appropriate to the real scene, with high visualization and clearer environmental information.

**Figure 20:**
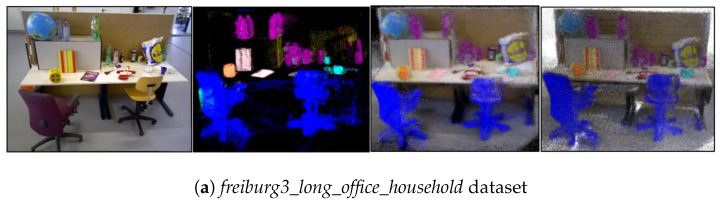

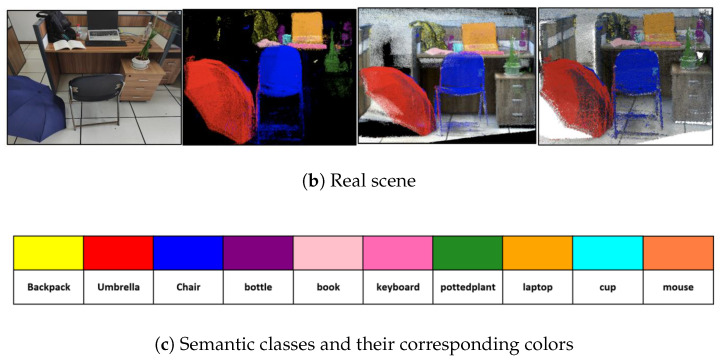
Semantic mapping effects of our algorithm on both the dataset and the real scene. (**a**,**b**) from left to right: the RGB image, the 3D dense map constructed only for the semantic entities obtained by YOLOv8-seg segmentation, the 3D dense semantic map of the scene, and the corresponding 3D semantic octree map.

To demonstrate the semantic mapping effect of the scene more clearly, we selected scene images from the front view of the table in both the *freiburg3_long_office_household* dataset and the real scene and leveraged the semantic information obtained from YOLOv8-seg to construct the 3D dense semantic map and corresponding 3D semantic octree map of the scene, as shown in Figure 20a,b. Moreover, to distinguish different categories of semantic entities, we pre-set 10 different colors to render entities according to the segmentation of entities in the scene, as shown in Figure 20c. We find that both in the dataset and in the real scene, the proposed algorithm was able to accurately recognize semantic entities in the scene; it could not only express semantic information clearly but also correctly integrate the pose relationships between entities into the semantic map. So the constructed map was consistent with the actual scene, without deformation or misalignment.

Through simulation and experimental results, it can be seen that the proposed vSLAM approach can enable mobile robots to achieve self-localization and mapping in complex dynamic environments. This provides reliable support for bionic robots to better mimic the perception and actions of organisms in natural environments. Meanwhile, combining the developed vSLAM system with software mechanisms provides a new perspective and effective method for the development of micro-bionic intelligent robots, which helps to better simulate the morphology and function of organisms. In addition, the application of our vSLAM technology in environmental perception and mapping also provides a good reference for biomimetics in understanding the perception and adaptation mechanisms of organisms with respect to the environment.

## 5. Conclusions and Future Work

This paper is concerned with the issue of the stability and accuracy degradation of SLAM algorithms in dynamic scenes due to the impact of moving objects or changes in scene structure, and it constructs a semantic vSLAM algorithm based on loop closure detection with combinatorial graph entropy. In the front end, the lightweight YOLOv8-seg and MDA were utilized to accurately detect and eliminate dynamic feature points. To prevent insufficient scene information caused by removing dynamic objects, a high-quality keyframe selection strategy was further developed. In the back end, according to the distribution of feature points, representation points, and semantic information, the unweighted and weighted graphs of keyframes were constructed, and then a high-performance loop closure detection method based on combinatorial graph entropy was constructed by calculating the graph entropy. A series of simulation and experimental results show that the proposed algorithm exhibited high accuracy and real-time performance in dynamic scenes, and the constructed maps were more in line with real scenes. Compared with ORB-SLAM2, the absolute trajectory accuracy in high-dynamic scenes mproved by an average of 96.86%, while the number of extracted keyframes decreased by an average of 61.20%. Compared with YOLOv5-based DO-SLAM, the segmentation and tracking time of our algorithm decreased by 68.95% and 76.74%, respectively. Also, our loop closure detection approach exhibited higher precision and recall in real scenes compared to the BoW model.

In future work, we intend to compensate for the segmentation network by introducing advanced mathematical methods or optical flow networks while ensuring real-time performance and preventing situations where dynamic objects are not recognized and where there are missed detections. In some special scenes, such as those with excessively bright or dim lighting, camera sensors may not be able to capture clear images. Since relying solely on RGB-D cameras as sensors may lead to insufficient acquired environmental information, we also plan to acquire richer environmental information through multi-sensor fusion to provide a better computational basis for the SLAM algorithm. In addition, we consider incorporating dynamic objects in semantic mapping to achieve tracking and reconstruction of dynamic scenes to better improve the adaptability of the algorithm. Indeed, addressing these aspects can enable bionic robots to better possess a human-like perception and better cope with environmental changes.

## Figures and Tables

**Table 1:** Experimental configurations.

Category	Configuration
Environment and libraries	C++, OpenCV3.4.10, Eigen3.3.7, Pangolin6.0 Sophus, G2O, Ceres2.0.0, DBoW1.8, PCL1.8.0, Octomap1.9.5
Wheeled mobile robot	Demo Board: jetson xavierNX (NVIDIA, Santa Clara, CA, USA) Depth vision sensor: RealSense D435i (Intel, Santa Clara, CA, USA) Driving mode: Two-wheel Differential Drive

**Table 2:** Comparison of RMSE for the ATE among different algorithms on the *TUM* dynamic dataset sequences (/m).

Sequences		RMSE (/m)						
		ORB- SLAM2	Dyna SLAM	Blitz- SLAM	MOLO- SLAM	Ji et al. [47]	Sun et al. [48]	Ours
High dynamic scenes	*w_xyz*	0.7615	0.0155	0.0153	0.015	0.0194	0.0150	**0.0148 **
	*w_half*	0.6215	0.0257	0.0256	0.0316	0.0290	0.0303	**0.0243**
	*w_rpy*	0.8545	0.0378	0.0356	0.0382	0.0371	0.0392	**0.0320**
	*w_static*	0.3340	0.0069	0.0102	**0.0060**	0.0111	0.0069	0.0078
Low dynamic scenes	*s_xyz*	**0.0091**	0.0159	0.0148	0.0109	0.0117	—	0.0111
	*s_half*	0.0242	0.0206	0.016	**0.0159**	0.0172	—	0.0218
	*s_rpy*	**0.0216**	—	—	—	—	—	0.0243
	*s_static*	0.0087	0.0108	—	—	—	0.0066	**0.0063**

**Table 3:** Comparison of RMSE for the RPE among different algorithms on *TUM* dynamic dataset sequences (/m).

Sequences		RMSE (/m)						
		ORB- SLAM2	Dyna SLAM	Blitz- SLAM	MOLO- SLAM	Ji et al. [47]	Sun et al. [48]	Ours
High-dynamic scenes	*w_xyz*	0.3884	0.0254	0.0197	0.0190	0.0234	0.0216	**0.0189**
	*w_half*	0.3772	0.0394	0.0253	0.0445	0.0423	0.0447	**0.0250**
	*w_rpy*	0.3838	0.0415	0.0473	0.0540	0.0471	0.0561	**0.0410**
	*w_static*	0.2056	0.0133	0.0129	**0.0080**	0.0117	0.0098	0.0106
Low-dynamic scenes	*s_xyz*	**0.0115**	0.0208	0.0144	0.0148	0.0166	—	0.0134
	*s_half*	0.0240	0.0306	0.0165	**0.0143**	0.0259	—	0.0229
	*s_rpy*	**0.0277**	—	—	—	—	—	0.0306
	*s_static*	0.0095	0.0126	—	—	—	0.0592	**0.0081**

**Table 4:** Comparison of the ATE for the ablation experiment of our algorithm under the f3_walking sequence.

Sequences	ORB-SLAM2		Ours (Y)		Ours (Y + M)		Ours (Y + M + K)	
	RMSE	SD	RMSE	SD	RMSE	SD	RMSE	SD
*w_xyz*	0.7615	0.4255	0.0159	0.008	0.0153	**0.0075**	**0.0148**	**0.0075**
*w_half*	0.6215	0.3134	0.0274	0.0123	0.0262	0.0123	**0.0243**	**0.0122**
*w_rpy*	0.8545	0.4393	0.0436	0.0303	0.0359	**0.0191**	**0.0320**	0.0196
*w-static*	0.3340	0.1444	0.0091	0.0041	0.0087	0.0044	**0.0078**	**0.0033**

**Table 5:** Comparison of time consumption among different algorithms.

Algorithm	ORB- SLAM2	Dyna- SLAM	DO- SLAM	YOLO- SLAM	Ours
Hardware platform	—	Nvidia Tesla M40 GPU	Inter Core i5-4288U	Intel Core i5-4288U CPU	Nvidia Geforce RTX 3050 GPU
Network	—	Mask R-CNN	YOLOv5	YOLOv3	YOLOv8-seg
Segmentation /Detection time (ms)	—	195	81.44	696.09	25.28
Tracking time (/ms)	22.8602	>300	118.23	651.53	26.7219

**Table 6:** Comparison of the results of different loop closure detection methods in different scenes.

Types of Scenes	Algorithm	Number of Closed-Loop	TP	FP	FN	Precision	Recall
Dynamic interference	BoW	8	6	0	2	100%	62.50%
	BoW+GE	8	8	0	0	100%	100%
Structural changes	BoW	8	7	0	1	100%	87.50%
	BoW+GE	8	8	0	0	100%	100%

## Data Availability

The raw data supporting the conclusions of this article will be made available by the authors on request.

## Abbreviations

The following abbreviations are used in this manuscript:

CGE-vSLAM	The proposed algorithm
vSLAM	Visual simultaneous localization and mapping
ORB	Oriented Fast and Rotated Brief
MAD	Mean Absolute Deviation
BOW	Bag of Words
BA	Bundle Adjustment
PCL	Point Cloud Library
ATE	Absolute Trajectory Error
RTE	Relative Translation Error
RMSE	Root Mean Square Error
